# Osteoclast-specific Plastin 3 knockout in mice fail to develop osteoporosis despite dramatic increased osteoclast resorption activity

**DOI:** 10.1093/jbmrpl/ziad009

**Published:** 2024-01-04

**Authors:** Ilka Maus, Maren Dreiner, Sebastian Zetzsche, Fabian Metzen, Bryony C Ross, Daniela Mählich, Manuel Koch, Anja Niehoff, Brunhilde Wirth

**Affiliations:** Institute of Human Genetics, University of Cologne, University Hospital of Cologne, 50931 Cologne, Germany; Center for Molecular Medicine Cologne, University of Cologne, 50931 Cologne, Germany; Institute of Biomechanics and Orthopaedics, German Sport University Cologne, 50933 Cologne, Germany; Institute of Human Genetics, University of Cologne, University Hospital of Cologne, 50931 Cologne, Germany; Center for Molecular Medicine Cologne, University of Cologne, 50931 Cologne, Germany; Medical Faculty, Institute for Dental Research and Oral Musculoskeletal Biology, University of Cologne, 50931 Cologne, Germany; Medical Faculty, Center for Biochemistry, University of Cologne, 50931 Cologne, Germany; Institute of Human Genetics, University of Cologne, University Hospital of Cologne, 50931 Cologne, Germany; Center for Molecular Medicine Cologne, University of Cologne, 50931 Cologne, Germany; Institute of Biomechanics and Orthopaedics, German Sport University Cologne, 50933 Cologne, Germany; Center for Molecular Medicine Cologne, University of Cologne, 50931 Cologne, Germany; Medical Faculty, Institute for Dental Research and Oral Musculoskeletal Biology, University of Cologne, 50931 Cologne, Germany; Medical Faculty, Center for Biochemistry, University of Cologne, 50931 Cologne, Germany; Institute of Biomechanics and Orthopaedics, German Sport University Cologne, 50933 Cologne, Germany; Faculty of Medicine, Cologne Center for Musculoskeletal Biomechanics (CCMB), University of Cologne, 50931 Cologne, Germany; Institute of Human Genetics, University of Cologne, University Hospital of Cologne, 50931 Cologne, Germany; Center for Molecular Medicine Cologne, University of Cologne, 50931 Cologne, Germany; Center for Rare Diseases, University of Cologne, University Hospital of Cologne, 50931 Cologne, Germany

**Keywords:** genetic animal model, osteoclasts, osteoporosis, Plastin 3, bone resorption

## Abstract

*PLS3* loss-of-function mutations in humans and mice cause X-linked primary osteoporosis. However, it remains largely unknown how *PLS3* mutations cause osteoporosis and which function PLS3 plays in bone homeostasis. A recent study showed that ubiquitous *Pls3* KO in mice results in osteoporosis. Mainly osteoclasts were impacted in their function However, it has not been proven if osteoclasts are the major cell type affected and responsible for osteoporosis development in ubiquitous *Pls3* KO mice. Here, we generated osteoclast-specific *Pls3* KO mice. Additionally, we developed a novel polyclonal PLS3 antibody that showed specific PLS3 loss in immunofluorescence staining of osteoclasts in contrast to previously available antibodies against PLS3, which failed to show PLS3 specificity in mouse cells. Moreover, we demonstrate that osteoclast-specific *Pls3* KO causes dramatic increase in resorptive activity of osteoclasts in vitro. Despite these findings, osteoclast-specific *Pls3* KO in vivo failed to cause any osteoporotic phenotype in mice as proven by micro-CT and three-point bending test. This demonstrates that the pathomechanism of PLS3-associated osteoporosis is highly complex and cannot be reproduced in a system singularly focused on one cell type. Thus, the loss of PLS3 in alternative bone cell types might contributes to the osteoporosis phenotype in ubiquitous *Pls3* KO mice.

## Teaser

Mutations in the *PLS3* gene that result in loss of function of the gene product are known to be responsible for the development of early-onset primary osteoporosis in humans and mice. Osteoporosis is a disease characterized by weakening, brittleness and loss of bones, as well as increased bone fragility and an increased risk of bone fractures. However, it is not clear how the mutations in *PLS3* cause osteoporosis and what function PLS3 plays in bone. Recently, it was shown that whole body deletion of *Pls3* in mice (ubiquitous *Pls3* knockout mice) leads to osteoporosis, characterized by increased bone thickness and stiffness in these mice. This revealed abnormalities in the bone cells that break down bone, called osteoclasts. In particular, their osteoclasts removed significantly more bone than osteoclasts from wild-type mice. Furthermore, osteoclast formation and activity, which are controlled by an important signaling pathway called the NF-κB pathway, were found to be abnormally regulated in the mice’s osteoclasts by whole-body deletion of *Pls3*. However, it remains unclear whether osteoclasts are the most affected cell type and are responsible for the development of osteoporosis in the mice with whole-body deletion of *Pls3*.

To find out whether osteoclast function was primarily affected by loss of *Pls3*, we specifically deleted the *Pls3* gene only in osteoclasts and generated so-called osteoclast-specific *Pls3* knockout KO mice. These mice only lack *Pls3* in their osteoclasts, while *Pls3* is still present and functional in the remaining cells and tissues. Remarkably, osteoclasts from these mice removed more bone than osteoclasts from normal mice. However, their bones did not show osteoporosis development in vivo in mice at 12, 24, and 48 wk of age, such as reduced bone strength and stiffness. These results suggest that deletion of *Pls3* from osteoclasts alone is not sufficient for the development of osteoporosis. Therefore, additional cells from the bones, such as the bone-forming cells, so-called osteoblasts, or the cells that control the activity of osteoclasts and osteoblasts, so-called osteocytes, could be further impaired in their function by the whole-body *Pls3* mice. Therefore, loss of *Pls3* from these additional bone cells could also contribute to the cause of osteoporosis. Furthermore, we successfully developed a first antibody against PLS3 to specifically visualize the presence of PLS3 in cells and tissues by immunofluorescence staining.

## Graphical Abstract

**Graphical Abstract:**
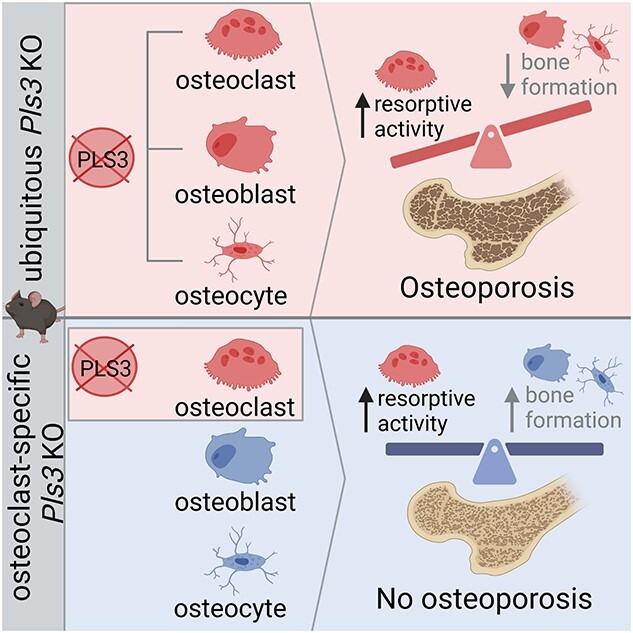

## Introduction

Osteoporosis is one of the most common skeletal disorders, affecting more than 200 million people worldwide.^1^ Due to the rising age of the common population, a high number of people, especially above the age of 50, is affected by osteoporosis.^2^ Thus, osteoporosis is associated with a high economic burden and is considered a public health issue.^3^^,^^4^ This systemic disease, affecting mostly postmenopausal women, Caucasians, and elderly people, is characterized by low BMD, reduced bone mass, and deteriorated bone microarchitecture, causing bone fragility and frequent fractures in the femoral neck, upper limbs, and body of the vertebrae with a T score (BMD score) of ≤ −2.5.^5–7^ Due to high treatment costs, disease prevention and therapy developments are of great importance.^8^

Osteoporosis results from the disequilibrium between bone-forming osteoblasts and bone-resorbing osteoclasts, where bone resorption exceeds bone formation.^1^^,^^9^ In this context, it has been shown that mutations in Plastin 3 (*PLS3*) are causative for BMD quantitative trait locus 18, BMND18; (MIM 300910), a monogenic X-linked, early-onset osteoporosis development in men and postmenopausal women, where the number of *PLS3* mutations identified is continuously rising.^10^^,^^11^

The gene encoding the highly conserved PLS3 protein is located on the X-chromosome.^10^^,^^12–14^ PLS3 is an F-actin–binding and F-actin–bundling protein and is ubiquitously expressed in all solid tissues.^12^^,^^15^ Interestingly, in 5% of healthy individuals, it is additionally detected in blood.^16^^,^^17^ PLS3 is expressed in almost all cell types of the bone, including osteoblasts, osteoclasts, osteocytes, and chondrocytes.^18–21^ Due to its important role in F-actin dynamics, it is essential for actin-dependent cellular processes including vesicle trafficking such as endocytosis and mechanotransduction. Furthermore, as mutations in *PLS3* resulted in reduced BMD, it is also suspected to play an essential role in bone metabolism.^10^^,^^17^^,^^22^ Moreover, it is speculated that PLS3 is important for Ca^2+^-dependent mechanotransduction in bone.^23^

However, it remains elusive, how *PLS3* mutations cause osteoporosis and which cellular pathways and cell systems are mainly involved. To elucidate the role of PLS3 in bone diseases, a ubiquitous *Pls3* KO mouse model has been generated to study the osteoporotic phenotype.^20^ Ubiquitous *Pls3* KO mice show an osteoporotic phenotype with decreased cortical and trabecular bone parameters and reduced strength and stiffness of the femur and vertebrae. Specifically, the osteoclasts are strongly affected by the loss of PLS3, exhibiting an increased resorptive activity, impaired podosome formation, as well as a disturbed NF-κB pathway, which has been hypothesized to be causative for the enhanced osteoclastogenesis in *Pls3* KO osteoclasts.^20^ Demonstrating these strong PLS3-dependent effects of osteoclast function, here we aim to unravel if the osteoclasts are the main cell type affected by *Pls3* loss and if a cell type–specific *Pls3* KO in osteoclasts is sufficient to cause a similar osteoporotic phenotype as detected in the ubiquitous *Pls3* KO mice. We generated an osteoclast-specific *Pls3* KO mouse model driven by LysMCre, which is expressed in the myeloid lineage, from which osteoclasts differentiate.^24^ This study aims to comprehensively characterize the osteoclasts and the bone phenotype of the generated mouse model and compare these findings to the ubiquitous *Pls3* KO mice to gain a deeper understanding of the role of PLS3 in the osteoporosis pathomechanism.

## Material and methods

### Mouse model—Ethical approval

All mice were housed in the animal facility of the CMMC (Centre for Molecular Medicine Cologne) under a 12-h light/dark cycle with access to food and water ad libitum. Breeding, housing, and experimental use of animals were performed in a specific pathogen-free environment. All animal experiments were approved by the Landesamt für Natur, Umwelt und Verbraucherschutz Nordrhein-Westfalen (LANUV) under the application number: 81-02.04.2020.A196.

### Pls3 KO mouse model

Ubiquitous *Pls3* KO mice were generated as described in Neugebauer et al.^20^ Briefly, the B6NTac;B6N-A^tm1Brd^*Pls3*^tm1a (EUCOMM)Wtsi/Wtsi^ (EMMA-ID: EM:06997, Wellcome Trust Sanger Institute) mouse line were purchased from the Wellcome Trust Sanger Institute and imported into the mouse facility through embryo transfer. Breedings, primers, allele names, and PCR conditions were applied as suggested by the manufacturer. To generate mice with a flipped out mutant allele (*tm1c*), where exon B of *Pls3* gene is flanked by loxP sites, the preconditional mice, containing the complete selection cassette *tm1a*, were crossbred with FLP-deleter mice.^25^ To generate ubiquitous *Pls3* KO mice, *tm1c*-expressing mice were then crossbred with ubiquitously expressing CMV-Cre-deleter mice^26^ to achieve excision of exon 2 of the *Pls3* gene (*tm1d*). Moreover, homo- and heterozygosity for females or males were checked by genotyping for either the WT or the mutant cassette. Genotyping for *tm1a*, *tm1c*, *tm1d*, and *Pls3* was performed as described by Neugebauer et al.^20^. Congenic WT C57BL/6 N mice were used as controls. All mice were held on a congenic C57BL/6 N background.

### Study design

The experimenter was blinded regarding genotypes of the mice at all steps until final statistical analysis. To perform micro-computed tomography (microCT) and three-point bending tests (3-PBTs), three different cohorts of animals including each genotype of males and females were sacrificed at 12, 24, and 48 wk of age, respectively. The exact number of animals used for each genotype, sex, and age is given in each figure legend and represented as dots in each graph. Experimenter was blinded regarding cell line genotypes during image acquisition, recordings, and statistical analysis. We conducted all experiments at least in triplicates. To avoid pseudo replication, the mean value per animal and mean value of technical replicates was applied for statistical analysis.

### Generation of osteoclast-specific Pls3 KO (Pls3^fl/fl^;LysMCre^tg/0^) mice

For the generation of osteoclast-specific *Pls3* KO (*Pls3*^fl/fl^;LysMCre^tg/0^) mice, the Cre-lox system was used. For this, conditional *Pls3* KO (*Pls3*^fl^) mice, carrying exon 2 of the *Pls3* allele flanked by loxP sites (*tm1c*), were crossbred with LysMCre (LysMCre^*+/*−^) mice.^24^ As LysM expression is restricted to cells of the myeloid lineage, *Pls3* will be specifically knocked-out in myeloid progenitor cells such as macrophages and subsequently osteoclasts. The cross-breeding resulted in the generation of *Pls3*^fl/fl^;LysMCre^+/−^ (osteoclast-specific *Pls3* KO) mice as well as their littermates *Pls3*^fl/fl^;LysMCre^−/−^. Genotyping of WT or mutant cassette was performed, and the following primers and PCR setups were used: Pls3_F: TACGCCATTACTCCCCATCC; Pls3_R: TTTCACACACTCGCCAAACAC; and Cas_R: TCGTGGTATCGTTATGCGCC. For the WT allele, the expected amplicon size was 581 bp, and for the mutant allele, the size was 311 bp. PCR conditions were 5 min 94 °C, 30 s 94 °C, 30 s 58 °C, 45 s 72 °C (repeat 34 cycles), and 5 min 72 °C. To check for the presence of heterozygous LysMCre, following primer setup and PCR conditions were applied: LysM_F: CTCTAGTCAGCCAGCAGCTG and LysM_R: ATGTTTAGCTGGCCCAAATGT. The expected amplicon size was 350 bp, and PCR conditions were similar to conditions described before. The *Pls3*^fl/fl^;LysMCre^−/−^ (called *Pls3*^fl/fl^) littermates were used as controls. All mice were held on a congenic C57BL/6 N background.

### Preparation of murine femora and spines

Preparation of femora and spines was performed as previously described.^20^ Briefly, mice were sacrificed, and the skin was removed around the hind limbs. To expose the hip joint, hind limb muscles and fascia were removed. With the forceps, the neck of the femur was grasped, and the hip joint was carefully dislocated by applying upward force, and attached ligaments were cut. The hip with femur and tibia was placed into DPBS, and the remaining skin and ligaments were carefully removed. Then the tibia was cut at the knee from the femur. For the spine isolation, the ribs were removed and the spine was carefully cleaned. The femur and the spine were wrapped in gauze tape, which was soaked in DPBS, and stored at −20 °C until analyzed.

### Micro-computed tomography

For nondestructive three-dimensional evaluation of bone microstructure in the femur, as well as in the spine, microCT measurements were performed as previously described using a Scanco μCT35 scanner (Scanco Medical AG, Bassersdorf, Switzerland).^20^^,^^27^ Briefly, scans of femora in 0.9% NaCl including protease inhibitors (Sigma-Aldrich, Steinheim, Germany) were captured at a voxel size of 7 × 7 × 7 μm, 70-kVp tube voltage, 114-μA tube current, and 400-ms integration time. To extract the trabecular bone, a global threshold of 22.0% was set. For the cortical bone, a threshold of 26.0% was applied. Trabecular bone was analyzed in the distal metaphysis in a volume situated 2000 –1000 μm proximal of the growth plate, which included only secondary spongiosa. For assessment of cortical bone, a 750 μm long volume was evaluated at the femoral midshaft. Trabecular parameters included bone volume fraction (BV/TV, %), trabecular thickness (Tb.Th, mm), trabecular number (Tb.N, 1/mm), trabecular separation (Tb.Sp, mm), and connectivity density (Conn.D, 1/mm^3^). At the diaphysis, cortical area (Ct.Ar, mm^2^), tissue area (Tt.Ar, mm^2^), cortical area fraction (Ct.Ar/Tt.Ar, %), cortical thickness (Ct.Th, mm), and marrow area (Ma.Ar, mm^2^) were assessed.

The microCT measurement at the spine was performed on the fourth, and fifth lumbar vertebrae (L4 and L5), as well as on the 12th and 13th thoracic vertebrae (T12 and T13). Scans of the spines in 0.9% NaCl were performed at a voxel size of 12 × 12 × 12 μm, 70-kVp tube voltage, 114-μA tube current, and 400-ms integration time. For the spine, 50% of the central body was assessed (± 25% up and down from the mid slice), segmenting only the trabecular structure, and the data were globally thresholded with 22.5%. We determined the same parameter as for trabecular bone at the femur. In addition, BMD (mgHA/cm^3^), cross-sectional area vertebral foramen (mm^2^), vertebral body height (mm), and the diameter of the vertebral foramen in anterior–posterior (A/P, mm) as well as medial-lateral (M/L, mm) direction were evaluated.

### Three-point bending test

Subsequently, femora were subjected to 3-PBT using a material testing machine (Z2.5/TN1S; Zwick; Germany) as previously described.^28^ Briefly, femora were loaded at mid-diaphysis in anterior–posterior direction using a 100-N load cell. The distance between support points, which had a diameter of 1.5 mm, was 5 mm. After preloading at 0.1 N, 0.05 mm/s, a loading rate of 1 mm/min was applied until failure. Breaking force (N) and deformation (mm), stiffness (N/mm), and energy (mJ) were measured from the load-deformation curve. Ultimate stress (MPa) and strain, elastic modulus (MPa), as well as energy density (mJ/mm^3^) were calculated based on the area moments of inertia obtained from microCT measurements.

### Radiographic studies and kyphosis index determination

Mice were sacrificed and placed on a radiographic cassette with a radiographic film. The exposure was done at 55 kV for 30 s. The radiographs were used to determine the kyphosis index (KI). For this, a line was drawn between the caudal margin of the last cervical vertebra to the caudal margin of the sixth lumbar vertebra, which corresponded to the cranial border of the wing of ilium. Then a line, placed perpendicular to this line, was drawn from the dorsal edge of the vertebra, where the curvature showed to be greatest. The KI was calculated by dividing the distance of the line between the vertebra by the distance of the line from this line to the curvature.^29^

### Osteoclast differentiation from spleen

Preparation of primary osteoclasts from spleen was performed as described by Boraschi-Diaz and Komarova.^30^ Briefly, spleens of 4-wk-old mice were aseptically removed, compressed using a plunger, and filtered through a strainer. After centrifugation, the pellet was dissolved in Red Cell Lysis Buffer, followed by two washing steps of the pellet by two centrifugation step in fresh medium. The cell pellet was resuspended in RPMI-1640 medium (ThermoFisher, #A10491091) + 10% FCS + penicillin/streptomycin. Differentiation of cells was initiated by the addition of 500 ng/mL M-CSF. On the next day, the culture medium was collected and centrifuged, and the pellet was resuspended in culture medium supplemented with M-CSF (250 ng/mL) (PeproTech, #315–02) and RANK-L (500 ng/mL) (PeproTech, #315–11) and added to the original culture flask. Cultures were maintained for 8 more days at 37 °C in an atmosphere of 5% CO_2_/air, and the medium, supplemented with M-CSF and RANKL, was replaced every 2–3 d.

### Primary cell cultures using murine embryonic fibroblasts

Primary murine embryonic fibroblasts (MEFs) were isolated and cultured as described.^31^

### Western blot analysis

Proteins from cell cultures were collected and lysed in RIPA buffer containing cOmplete mini protease inhibitor cocktail (Sigma-Aldrich, #11836153001). Protein concentrations were measured using Bradford reagent. Western blot analysis was performed according to standard protocols. For analysis, the following primary antibodies were used: anti-PLS3 (Eurogentec [^17^; 1238], anti-N-terminal PLS3 [this manuscript; 3772], anti-PLS3 [Human Protein Atlas, HPA020433], and anti-beta-actin HRP-conjugated (Proteintech Cat# HRP-60008). Signal was detected with rabbit-HRP-conjugated secondary antibody (Cell Signaling Technology Cat# 7074). ACTB was used as internal standard, and the chemiluminescence reagent was used to develop the immunosignals (Thermo Scientific).

### Development of an immunofluorescence staining–competent and specific PLS3 antibody

To generate a PLS3-specific antibody, we expressed an 87 AA stretch with the lowest homologies to plastin-1 (64% identities) and to plastin-2 (54% identities). This region (*Mus musculus*; NP_001159925; AA4..AA90) was expressed with a BM40 signal peptide, a Twin-Strep-tag®, and a carrier protein. Prior to the immunization, the Twin-Strep-tag® was removed by thrombin digestion. Recombinant PLS3 in PBS was used to immunize two rabbits (Davids Biotechnologie GmbH). After 2 mo, sera were collected, and the antibodies affinity purified via a PLS3 affinity column. Tag-free PLS3 was coupled to CNBr-activated Sepharose (GE Healthcare), and after applying the sera to the column, the specific antibodies were eluted with 150 mM NaCl, 0.1 M glycine–HCl, pH 2.5, and immediately neutralized with 1 M Tris–HCl, pH 8, then dialyzed against PBS. The two antibodies were tested and validated by Western blot analysis and immunohistochemistry.

### Immunohistochemistry

For immunofluorescence staining, differentiated osteoclasts and MEFs were fixed with 4% PFA and incubated with primary antibody at 4 °C O/N. Incubation of the secondary antibody was performed for 2 h at RT in the dark. The following antibodies were used: anti-N-terminal PLS3 1: 300; Phalloidin, AlexaFluor 647 1:400 (Thermo Fisher Scientific, #A22287); anti-PLS3 1:200 (^17^ Eurogentec, 1238); anti-PLS3 1:200 (Human Protein Atlas, HPA020433); Hoechst (NucBlue) 1 drop/500 μL (Thermo Fisher Scientific, #R37605); and rabbit AlexaFluor 488 1:300 (Thermo Fisher Scientific, #A21206). Cells were mounted in Mowiol (Sigma, #81381).

### Image acquisition and analysis

Fluorescence images and immunofluorescence stainings were acquired with Zeiss microscope (AxioImager.M2) supplied with the Apotome.2 system to mimic a confocal microscope. Images were acquired as Z-stacks 40× oil objective.

### Resorption pit assay

Resorption pit assay was performed as previously described.^20^ Osteoclast precursor cells from spleen-derived macrophages were differentiated into mature osteoclasts for 10 d in RPMI-1640 medium with the supplementation of 250 ng/μm M-CSF and 500 ng/μL RANK-L. Afterward, 250 000 osteoclasts/mL were transferred onto bovine bone slice (boneslices.com), placed in 96-well plates, and allowed to resorb bone for 7 d with the regular exchange of medium supplemented with M-CSF and RANK-L. For the staining of resorption pits, cells were removed by ultrasonication, followed by toluidine blue (Sigma-Aldrich, #T3260) staining. The quantification of the resorption pit area was conducted with the ZEN software (RRID:SCR_013672) (Zeiss).

### Statistics

Statistical analyses were performed using the software programs GraphPad Prism 9 (RRID:SCR_002798) and Excel2013 (RRID:SCR_016137) (Microsoft). For equal variances, two-tailed Student *t*-tests was used; for unequal variances of two independent groups, the Mann–Whitney U-test was applied. The logarithmic resorbed area was evaluated by two-way ANOVA with factors genotype and animal nested within genotype. To guard against type-I-error inflation in pairwise comparisons, Tukey’s HSD method was applied. Notable, the corresponding inference space is narrow,^32^ i.e. inferences pertain to the specific sample of animals. Specific tests, sample size, data representation, and *P*-values are indicated in figure legends. Significance thresholds were: *P* < .05^*^, *P* < .01^*^^*^, *P* < .001^*^^*^^*^, and ns = not significant. As indicated, the data are presented as mean ± SEM or as mean ± SD. The box plots show all individual data points with the median as a line, interquartile range (25th–75th percentile), and min to max as whiskers.

## Results

### Generation of osteoclast-specific Pls3 KO mice

We previously demonstrated that the ubiquitous *Pls3* KO in mice results in osteoporosis and strongly impairs the osteoclast function.^20^ To reveal if the osteoclasts are the major and solely cell system affected by the loss of *Pls3* and are responsible for the development of osteoporosis, we generated an osteoclast-specific *Pls3* KO mouse line. To achieve osteoclast-specific *Pls3* KO, conditional *Pls3*-floxed mice (*Pls3*^fl/fl^) were crossbred with LysMCre^tg/0^ mice, resulting in the specific deletion of *Pls3* in cells of the myeloid lineage, which give rise to osteoclasts during development.^24^ Additional crossbreedings for three generations were conducted to result in the generation of 50% female *Pls3*^fl/fl^;LysMCre^tg/0^ or male *Pls3*^fl^;LysMCre^tg/0^ mice, defined as osteoclast-specific *Pls3* KO mice, and 50% female *Pls3*^fl/fl^ or male *Pls3*^fl^ mice, defined as *Pls*3-floxed mice. Genotyping was used to differentiate *Pls3*^fl/fl^;LysMCre^tg/0^ or *Pls3*^fl^;LysMCre^tg/0^ from *Pls3*^fl/fl^ or *Pls3*^fl^ mice and confirmed the successful osteoclast-specific *Pls3* KO for the deleted and the *Pls3*-floxed allele (Supplemental Figure S1A).

The osteoclast-specific *Pls3* KO mice were viable and did not show weight differences compared to *Pls3-*floxed mice (data not shown). To assess bone-specific morphological changes of the spine, the KI^29^ was determined in 12-wk-old female and male osteoclast-specific *Pls3* KO mice in comparison to *Pls*3-floxed littermates and WT mice. For both newly generated mouse groups, no kyphosis phenotype was identified in comparison to WT mice (Supplemental Figure S1B). Since both female and male *Pls3*-floxed mice were unaffected compared to WT mice, these were used as control littermates for further studies.

### Characterization of osteoclasts

We hypothesize that PLS3 plays an important role in osteoclast differentiation and bone resorption due to the previously identified effect on the osteoclasts’ resorptive activity, podosome formation, and NF-κB pathway in ubiquitous *Pls3* KO mice.^20^

**Figure 1:**
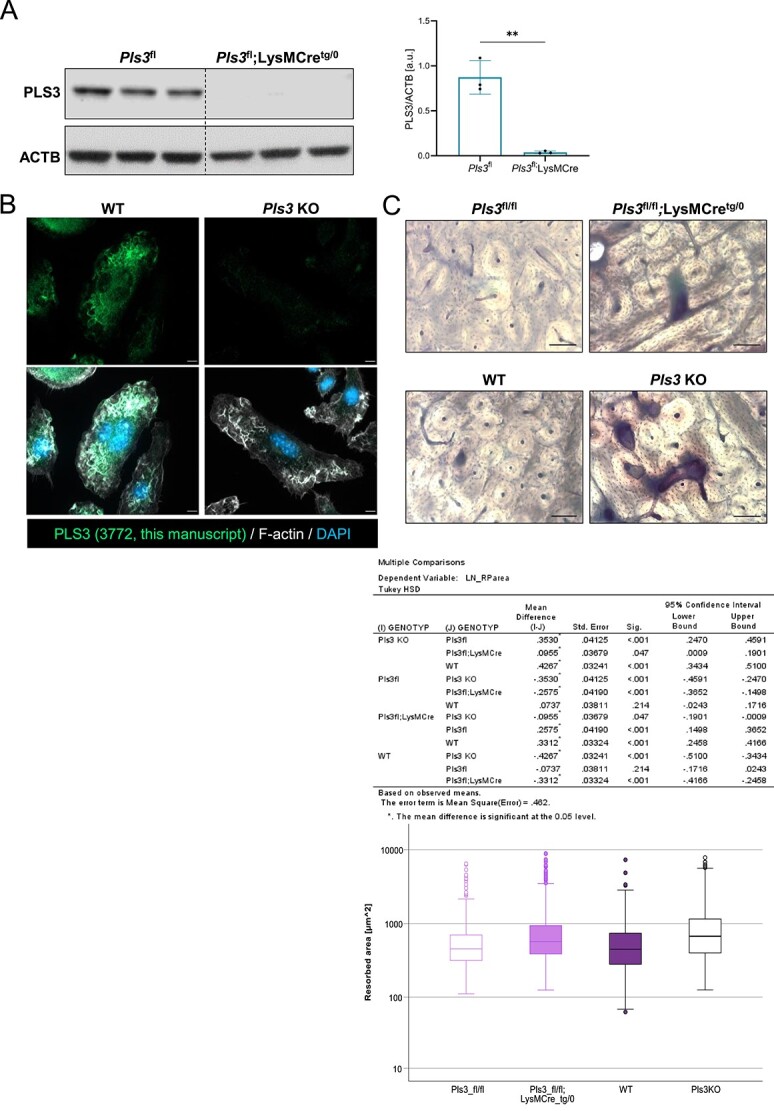
Characterization of *Pls3^fl/fl^;LysMCre^tg/0^* osteoclasts and validation of the specificity of a newly developed polyclonal PLS3 antibody. (A) Protein levels of PLS3 and the housekeeper ACTB in male *Pls3^fl^* and *Pls3^fl^;LysMCre^tg/0^* osteoclasts. Quantification of PLS3 protein levels relative to ACTB confirmed the osteoclast-specific Pls3 deletion in the osteoclasts of these mice. N = 3. Results are shown as mean ± SD. Statistical test: Unpaired two-tailed Student’s t-test **p < 0.01 (B) immunofluorescence staining of differentiated WT control and *Pls3* KO osteoclasts with a newly developed PLS3 antibody (this manuscript, 3772) confirmed antibody specificity. PLS3 staining resulted in filamentous structures in WT control osteoclasts, whereas no signal was detected in Pls3 KO osteoclasts. The actin cytoskeleton was stained with F-actin and nuclei were stained with DAPI. (C) Resorption pit assay of primary spleen-derived osteoclasts from 1-mo-old mice cultivated on bovine bone slices for 7 d and stained with toluidine blue. Resorbed areas of Pls3^fl/fl^, Pls3^fl/fl^;LysMCre^tg/0^, WT and, Pls3 KO were compared to each other. Scale bar: 10 μm. N = 3-7 animals per genotype, n > 90 resorption pits per animal. Results of multiple comparison analyses are shown in the table below and in the graphical representation beneath. Statistical test: Log resorbed area was evaluated by two-way ANOVA with factors genotype and animal nested within genotype. To guard against type-I-error inflation in pairwise comparisons, Tukey‘s HSD method was applied. Notable, the corresponding inference space is narrow,^32^ i.e. inferences pertain to the specific sample of animals.

To demonstrate that *Pls3* was specifically and successfully deleted in osteoclasts of osteoclast-specific *Pls3* KO mice, spleen-derived primary differentiated osteoclasts were cultured and protein lysates were analyzed by Western blotting. PLS3 protein was absent in the differentiated osteoclast-specific *Pls3* KO osteoclasts (Figure 1A).

Besides, we demonstrated that PLS3 abundancy was equally absent or present in osteoclasts derived from bone marrow of 12-wk-old osteoclast-specific *Pls3* KO mice and their *Pls3*-floxed controls, compared to spleen-derived osteoclasts from ubiquitous *Pls3* KO or osteoclast-specific *Pls3* KO mice as compared to WT mice (Figure 1A, Supplemental Figure S1C–D).

Based on these data and because the number of osteoclasts derived from precursor cells from spleen was higher than from bone marrow, we were not only able to reduce the number of animals but also to conduct our experiments much faster and to lower costs.

Moreover, we performed immunofluorescence staining of osteoclasts with a newly developed polyclonal PLS3 antibody, which showed no signals in the *Pls3* deleted but in WT control osteoclasts (Figure 1B). Importantly to note, our old polyclonal PLS3 antibody developed by Eurogentec (#1238),^17^ as well as other antibodies available on the market (anti-PLS3 Antibody from Human Protein Atlas [HPA020433]), failed to show PLS3-specific signals in MEFs in contrast to the newly developed PLS3 antibody (this manuscript, 3772) (Supplemental Figure S2A–C). Nonetheless, the former PLS3 antibody from Eurogentec and the newly developed one, but not from Human Protein Atlas, are specific for Western blots (Supplemental Figure S2D–F).

As resorptive activity of osteoclasts from ubiquitous *Pls3* KO mice was strongly affected,^20^ we conducted resorption pit assay with osteoclast-specific *Pls3* KO osteoclasts. In addition, we included osteoclasts from WT and ubiquitous *Pls3* KO mice to directly compare the results to the osteoclast-specific *Pls3* KO mice.

Osteoclasts from osteoclast-specific *Pls3* KO mice produced abundant and notably increased resorption pit areas, exhibiting a 49% increase (1.5-fold increase) in comparison to their control littermates (Figure 1C). In line with previous results,^20^ ubiquitous *Pls3* KO osteoclasts showed a 65% increase (1.7-fold increase) in resorption pit area and thus an enhanced resorptive activity compared to the WT controls. The strong increase in resorptive activity of osteoclast-specific *Pls3* KO and ubiquitous *Pls3* KO osteoclasts occurred likewise when comparing to *Pls3*-floxed or WT osteoclasts; there was no difference between *Pls3*-floxed and WT osteoclasts resorptive pit areas (Figure 1C). These results demonstrate the successful generation of osteoclast-specific *Pls3* KO mice and confirm that *PLS3* regulates the resorptive activity in osteoclasts.

### Absence of Pls3 in osteoclasts does not cause osteoporosis in the femur

Increased bone resorptive activity of osteoclasts is known to directly influence the bone quality, leading to a weakening of the bone microarchitecture and decreasing bone strength and stiffness, and might subsequently result in osteoporosis.^33^^,^^34^

To evaluate the influence of the osteoclast-specific *Pls3* KO on bone morphology, the trabecular and cortical bone microstructure of femora and spines were analyzed by microCT, and the mechanical properties of the femora were examined by 3-PBT. To this end, the same bone morphometric measurements previously performed in 12-wk-old ubiquitous *Pls3* KO mice were applied on osteoclast-specific *Pls3* KO mice and their control littermates, and the measurements were compared.^20^ Female and male mice were analyzed separately, to detect possible sex differences characteristic for osteoporosis.

**Figure 2:**
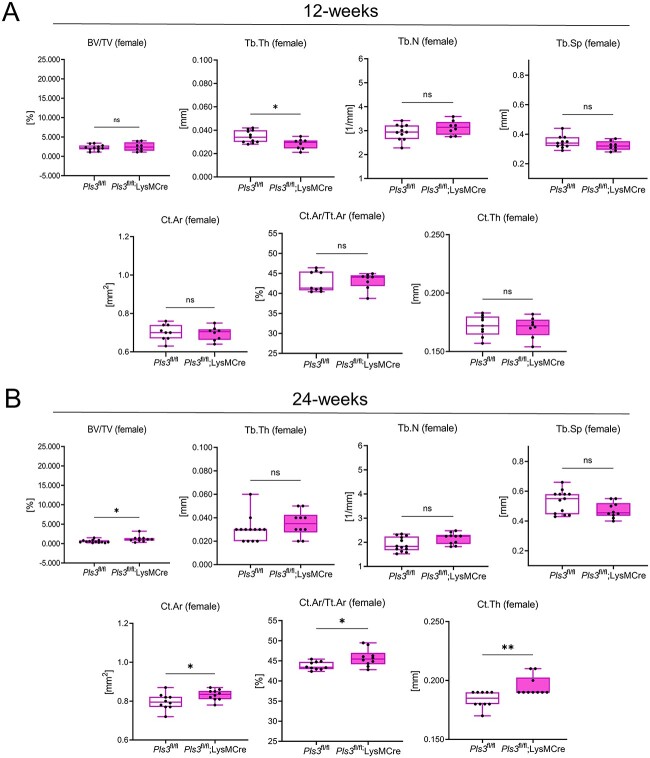
Selected microCT data of the femora from 12- and 24-wk-old female *Pls3*^fl/fl^;*LysM*Cre^tg/0^ mice in comparison to their *Pls3*^fl/fl^ littermates. Shown are bone volume fraction (BV/TV, %), trabecular thickness (Tb.Th, mm), trabecular number (Tb.N, 1/mm), trabecular separation (Tb.Sp, mm), cortical area (Ct.Ar, mm^2^), ratio of cortical area to tissue area (Ct.Ar/Tt.Ar, %), and cortical thickness (Ct.Th, mm) for (A) 12-wk-old female mice and (B) for 24-wk-old female mice. *N* = 8 of 12-wk-old female *Pls3*^fl/fl^;*LysM*Cre^tg/0^ mice, *N* = 11 of 12-wk-old female *Pls3*^fl/fl^ mice; *N* = 10 of 24-wk-old female *Pls3*^fl/fl^;*LysM*Cre^tg/0^ mice; *N* = 13 of 24-wk-old female *Pls3*^fl/fl^ mice. All results are shown as box plots, representing individual data points with median as a line, interquartile range (25th–75th percentile), and min to max as whiskers. ^*^P < .05, ^*^^*^P < .01, ^*^^*^^*^P < .001, ns = not significant. Statistical test: Mann–Whitney U test.

No marked changes in trabecular parameters of 12-wk-old female and male osteoclast-specific *Pls3* KO mice were measured, except of a significant decrease in the trabecular thickness (Tb.Th) (18.3%, *P* < .01) in female osteoclast-specific *Pls3* KO mice (Figure 2A, Supplemental Figure S3A, Table 1). In contrast, for female and male ubiquitous *Pls3* KO mice, the Tb.Th was unaffected, but the trabecular number (Tb.N) and connectivity density (Conn.D) were significantly decreased, and the trabecular separation (Tb.Sp) significantly increased (Table 1, first column).^20^

**Table 1:** microCT data of the femora from ubiquitous *Pls3* KO mice compared to osteoclast-specific *Pls3* KO mice.

microCT of femur		12 wk		24 wk	48 wk
		*Pls3* KO vs WT	*Pls3* ^fl/fl^;LysMCre vs *Pls3*^fl/fl^	*Pls3* ^fl/fl^;LysMCre vs *Pls3*^fl/fl^	*Pls3* ^fl/fl^;LysMCre vs *Pls3*^fl/fl^
		Neugebauer et al.^20^	This study		
BV/TV (%)	Female	^*^^*^ decrease	ns	^*^ increase	ns
	Male	ns	ns	ns	ns
Tb.Th (mm)	Female	ns	^*^^*^ decrease	ns	ns
	Male	ns	ns	ns	ns
Tb.N (1/mm)	Female	^*^^*^^*^ decrease	ns	ns	ns
	Male	^*^^*^^*^ decrease	ns	ns	ns
Tb.Sp (mm)	Female	^*^^*^^*^ increase	ns	ns	ns
	Male	^*^^*^^*^ increase	ns	ns	ns
Conn.D (1/mm^3^)	Female	^*^ decrease	ns	^*^ increase	ns
	Male	^*^ decrease	ns	ns	ns
Ct.Ar (mm^2^)	Female	^*^^*^^*^ decrease	ns	^*^ increase	ns
	Male	^*^ decrease	ns	ns	ns
Tt.Ar (mm^2^)	Female	^*^ increase	ns	ns	ns
	Male	ns	ns	ns	ns
Ct.Ar/Tt.Ar (%)	Female	^*^^*^^*^ decrease	ns	^*^ increase	ns
	Male	^*^^*^^*^ decrease	ns	ns	ns
Ct.Th (mm)	Female	^*^^*^^*^ decrease	ns	^*^^*^ increase	ns
	Male	^*^^*^^*^ decrease	ns	ns	ns
Ma.Ar (mm^2^)	Female	^*^^*^ increase	ns	ns	ns
	Male	^*^^*^ increase	ns	ns	ns

Previously, trabecular and cortical bone parameters, measured by microCT, were determined in 12-wk-old female and male ubiquitous *Pls3* KO mice and compared to WT mice.^20^ In this study, similar parameters were determined for femora of 12-, 24-, and 48-wk-old osteoclast-specific *Pls3* KO female and male mice in comparison to their *Pls3*-floxed control littermates. Shown are ratio of bone volume fraction (BV/TV, %), trabecular thickness (Tb.Th, mm), trabecular number (Tb.N, 1/mm), trabecular separation (Tb.Sp, mm), connectivity density (Conn.D, 1/mm^3^), cortical area (Ct.Ar, mm^2^), total area (Tt.Ar, mm^2^), ratio of Ct.Area to tissue area (Ct.Ar/Tt.Ar, %) ratio, cortical thickness (Ct.Th, mm), and marrow area (Ma.Ar, mm^2^). *N* = 10 of 12-wk-old ubiquitous *Pls3* KO mice; *N* = 10 of 12-wk-old WT mice; *N* = 8 of 12-wk-old female *Pls3*^fl/fl^;LysMCre^tg/0^ mice; *N* = 11 of 12-wk-old female *Pls3*^fl/fl^ mice; *N* = 12 of 12-wk-old male P*ls3*^fl^;LysMCre^tg/0^ mice; *N* = 11 of 12-wk-old male *Pls3*^fl^ mice; *N* = 10 of 24-wk-old female *Pls3*^fl/fl^;LysMCre^tg/0^ mice; *N* = 13 of 24-wk-old female *Pls3*^fl/fl^ mice. *N* = 13 of 24-wk-old male *Pls3*^fl^;LysMCre^tg/0^ mice; *N* = 9–11 of 24-wk-old male *Pls3*^fl^ mice; *N* = 5 of 48-wk-old female *Pls3*^fl/fl^;LysMCre^tg/0^ mice; *N* = 3–8 of 48-wk-old female *Pls3*^fl/fl^ mice; *N* = 9 of 48-wk-old male *Pls3*^fl^;LysMCre^tg/0^ mice; *N* = 9 of 48-wk-old male *Pls3*^fl^ mice. All results are shown as mean ± SEM. ^*^P < .05, ^*^^*^P < .01, ^*^^*^^*^P < .001, ns = not significant. An increase or decrease in comparison to the control group is indicated. Statistical test: Mann–Whitney U test.

Similarly, all cortical parameters measured in femora of 12-wk-old female and male osteoclast-specific *Pls3* KO mice remained unaffected compared to their control littermates (Figure 2A, Supplemental Figures S3 and S4A, Table 1). These findings did not resemble the osteoporotic phenotype, detected in the femora of aged- and sex-matched ubiquitous *Pls3* KO mice, which showed significant changes in cortical parameters compared to the WT controls (Table 1, first column).^20^

Consequently, we included 24- and 48-wk-old female and male osteoclast-specific *Pls3* KO and control littermates in our study to test whether phenotypic changes develop at a later time-point. Similar to the results in the 12-wk-old osteoclast-specific *Pls3* KO mice, a bone phenotype was absent in 24-wk-old female and male mice (Figure 2B, Supplemental Figure S4B, Table 1). Opposing to the results obtained in 12-wk-old ubiquitous *Pls3* KO mice, an increase in the BV/TV (103.1%, *P* < .05), Conn.D (116.3%, *P* < .05), Ct.Ar (4.5%, *P* < .05), Ct.Ar/Tt.Ar (4.5%, *P* < .05), and Ct.Th (5.4%, *P* < .01) was detected for female 24-wk-old osteoclast-specific *Pls3* KO mice (Figure 2B, Supplemental Figure S4B, Table 1). Unexpectedly, these changes were not detected in 48-wk-old female and male osteoclast-specific *Pls3* KO mice compared to control littermates (Supplemental Figures S3 and S4C, Table 1).

### Absence of Pls3 in osteoclasts does not cause osteoporosis in the spine

Additionally, *Pls3*-dependent changes in the spine were investigated by microCT measurements in 12-wk-old female and male osteoclast-specific *Pls3* KO mice, focusing on the trabecular bone of the lumbar regions L4 and L5, and the thoracic regions T12 and T13 (Supplemental Figures S5–12, Tables 2 and 3).

**Table 2:** microCT data of the vertebrae L4 in 12-, 24-, and 48-wk-old female and male osteoclast-specific *Pls3* KO mice in comparison to their *Pls3*-floxed control littermates.

microCT of spine L4		12 wk	24 wk	48 wk
		*Pls3* ^fl/fl^;LysMCre vs *Pls3*^fl/fl^	*Pls3* ^fl/fl^;LysMCre vs *Pls3*^fl/fl^	*Pls3* ^fl/fl^;LysMCre vs *Pls3*^fl/fl^
BV/TV (%)	Female	ns	ns	ns
	Male	ns	ns	ns
BMD (mgHA/cm^3^)	Female	ns	ns	ns
	Male	ns	ns	ns
Tb.N (1/mm)	Female	ns	ns	ns
	Male	ns	ns	ns
Tb.Sp (mm)	Female	ns	ns	ns
	Male	ns	ns	ns
Tb.Th (mm)	Female	ns	^*^ increase	ns
	Male	ns	ns	ns
Conn.D (1/mm^3^)	Female	ns	ns	ns
	Male	ns	ns	ns
Vertebral foramen A/P (mm)	Female	ns	^*^ increase	ns
	Male	ns	ns	ns
Vertebral foramen M/L (mm)	Female	ns	ns	ns
	Male	ns	ns	ns
Cross-sectional area vertebral foramen (mm^2^)	Female	ns	ns	ns
	Male	ns	ns	ns
Vertebral body height (mm)	Female	ns	ns	ns
	Male	ns	ns	ns

Shown are bone volume fraction (BV/TV, %), BMD (mmHA/cm^3^), trabecular number (Tb.N, 1/mm), trabecular separation (Tb.Sp, mm), trabecular thickness (Tb.Th, mm), and connectivity density (Conn.D, 1/mm^3^), vertebral foramen A/P (mm), vertebral foramen M/L (mm), cross-sectional area of the vertebral foramen (mm^2^), and vertebral body height (mm). *N* = 8 of 12-wk-old female *Pls3*^fl/fl^;LysMCre^tg/0^ mice; *N* = 12 of 12-wk-old female *Pls3*^fl/fl^ mice; *N* = 12 of 12-wk-old male *Pls3*^fl^;LysMCre^tg/0^ mice; *N* = 11 of 12-wk-old male *Pls3*^fl^ mice; *N* = 11 of 24-wk-old female *Pls3*^fl/fl^;LysMCre^tg/0^ mice; *N* = 13 of 24-wk-old female *Pls3*^fl/fl^ mice; *N* = 13 of 24-wk-old male *Pls3*^fl^;LysMCre^tg/0^ mice; *N* = 11 of 24-wk-old male *Pls3*^fl^ mice; *N* = 3 of 48-wk-old female *Pls3*^fl/fl^;LysMCre^tg/0^ mice; *N* = 5 of 48-wk-old female *Pls3*^fl/fl^ mice; *N* = 5 of 48-wk-old male *Pls3*^fl^;LysMCre^tg/0^ mice; *N* = 4 of 48-wk-old male *Pls3*^fl^ mice. All results are shown as mean ± SEM. ^*^P < .05, ^*^^*^P < .01, ^*^^*^^*^P < .001, ns = not significant. An increase or decrease in comparison to the control group is indicated. Statistical test: Mann–Whitney U test.

**Table 3:** microCT data of the vertebrae L5 in 12-, 24-, and 48-wk-old female and male osteoclast-specific *Pls3* KO mice in comparison to their *Pls3*-floxed control littermates.

microCT of spine L5		12 wk	24 wk	48 wk
		*Pls3* ^fl/fl^;LysMCre vs *Pls3*^fl/fl^	*Pls3* ^fl/fl^;LysMCre vs *Pls3*^fl/fl^	*Pls3* ^fl/fl^;LysMCre vs *Pls3*^fl/fl^
BV/TV (%)	Female	ns	ns	ns
	Male	ns	ns	ns
BMD (mgHA/cm^3^)	Female	ns	ns	ns
	Male	ns	ns	ns
Tb.N (1/mm)	Female	ns	ns	ns
	Male	^*^^*^ increase	ns	ns
Tb.Sp (mm)	Female	ns	ns	ns
	Male	^*^ decrease	ns	ns
Tb.Th (mm)	Female	ns	ns	ns
	Male	ns	ns	ns
Conn.D (1/mm^3^)	Female	ns	ns	ns
	Male	ns	ns	ns
Vertebral foramen A/P (mm)	Female	ns	ns	ns
	Male	ns	ns	ns
Vertebral foramen M/L (mm)	Female	ns	ns	ns
	Male	ns	ns	ns
Cross-sectional area vertebral foramen (mm^2^)	Female	ns	ns	ns
	Male	ns	ns	ns
Vertebral body height (mm)	Female	ns	ns	ns
	Male	ns	ns	ns

Shown are bone volume fraction (BV/TV, %), BMD (mgHA/cm^3^), trabecular number (Tb.N, 1/mm), trabecular separation (Tb.Sp, mm), trabecular thickness (Tb.Th, mm), and connectivity density (Conn.D, 1/mm^3^), vertebral foramen A/P (mm), vertebral foramen M/L (mm), cross-sectional area of the vertebral foramen (mm^2^), and vertebral body height (mm). *N* = 8 of 12-wk-old female *Pls3*^fl/fl^;LysMCre^tg/0^ mice; *N* = 12 of 12-wk-old female *Pls3*^fl/fl^ mice; *N* = 12 of 12-wk-old male *Pls3*^fl^;LysMCre^tg/0^ mice; *N* = 11 of 12-wk-old male *Pls3*^fl^ mice; *N* = 11 of 24-wk-old female *Pls3*^fl/fl^;LysMCre^tg/0^ mice; *N* = 13 of 24-wk-old female *Pls3*^fl/fl^ mice; *N* = 13 of 24-wk-old male *Pls3*^fl^;LysMCre^tg/0^ mice; *N* = 11 of 24-wk-old male *Pls3*^fl^ mice; *N* = 3 of 48-wk-old female *Pls3*^fl/fl^;LysMCre^tg/0^ mice; *N* = 5 of 48-wk-old female *Pls3*^fl/fl^ mice; *N* = 5 of 48-wk-old male *Pls3*^fl^;LysMCre^tg/0^ mice; *N* = 4 of 48-wk-old male *Pls3*^fl^ mice. All results are shown as mean ± SEM. ^*^P < .05, ^*^^*^P < .01, ^*^^*^^*^P < .001, ns = not significant. An increase or decrease in comparison to the control group is indicated. Statistical test: Mann–Whitney U test.

MicroCT analysis of vertebrae L4 and L5 did not reveal any significant differences in the microstructure for female and male osteoclast-specific *Pls3* KO mice, except of a significant increase in the Tb.N (6.0%, *P* < .01) and a decrease in the Tb.Sp (6.9%, *P* < .05) in male osteoclast-specific *Pls3* KO mice for L5 (Supplemental Figures S5–S8, Tables 2 and 3).

For the vertebrae T12, the vertebral foramen M/L (3.5%, *P* < .01) and cross-sectional area of the vertebral foramen (4.8%, *P* < .05) were markedly increased in male osteoclast-specific *Pls3* KO mice (Supplemental Figure S10A, Table 4). Moreover, for the vertebrae T13, an increase in the vertebral foramen M/L (2.1%, *P* < .05) and vertebral body height (2.8%, *P* < .05) was measured in 12-wk-old male osteoclast-specific *Pls3* KO mice (Supplemental Figure S12A, Table 5). Remaining parameters were unaffected (Supplemental Figures S9–12A, Tables 4 and 5).

**Table 4:** microCT data of the vertebrae T12 in 12-, 24-, and 48-wk-old female and male osteoclast-specific *Pls3* KO mice in comparison to their *Pls3*-floxed control littermates.

microCT of spine T12		12 wk	24 wk	48 wk
		*Pls3* ^fl/fl^;LysMCre vs *Pls3*^fl/fl^	*Pls3* ^fl/fl^;LysMCre vs *Pls3*^fl/fl^	*Pls3* ^fl/fl^;LysMCre vs *Pls3*^fl/fl^
BV/TV (%)	Female	ns	ns	ns
	Male	ns	ns	ns
BMD (mgHA/cm^3^)	Female	ns	ns	ns
	Male	ns	ns	ns
Tb.N (1/mm)	Female	ns	ns	ns
	Male	ns	ns	ns
Tb.Sp (mm)	Female	ns	ns	ns
	Male	ns	ns	ns
Tb.Th (mm)	Female	ns	ns	ns
	Male	ns	ns	ns
Conn.D (1/mm^3^)	Female	ns	ns	^*^ increase
	Male	ns	ns	ns
Vertebral foramen A/P (mm)	Female	ns	ns	ns
	Male	ns	ns	ns
Vertebral foramen M/L (mm)	Female	ns	ns	ns
	Male	^*^^*^ increase	ns	ns
Cross-sectional area vertebral foramen (mm^2^)	Female	ns	ns	ns
	Male	^*^ increase	ns	ns
Vertebral body height (mm)	Female	ns	ns	ns
	Male	ns	ns	ns

Shown are bone volume fraction (BV/TV, %), BMD (mgHA/cm^3^), trabecular number (Tb.N, 1/mm), trabecular separation (Tb.Sp, mm), trabecular thickness (Tb.Th, mm), and connectivity density (Conn.D, 1/mm^3^), vertebral foramen A/P (mm), vertebral foramen M/L (mm), cross-sectional area of the vertebral foramen (mm^2^), and vertebral body height (mm). *N* = 8 of 12-wk-old female *Pls3*^fl/fl^;LysMCre^tg/0^ mice; *N* = 12 of 12-wk-old female *Pls3*^fl/fl^ mice; *N* = 12 of 12-wk-old male *Pls3*^fl^;LysMCre^tg/0^ mice; *N* = 11 of 12-wk-old male *Pls3*^fl^ mice; *N* = 11 of 24-wk-old female *Pls3*^fl/fl^;LysMCre^tg/0^ mice; *N* = 13 of 24-wk-old female *Pls3*^fl/fl^ mice; *N* = 13 of 24-wk-old male *Pls3*^fl^;LysMCre^tg/0^ mice; *N* = 11 of 24-wk-old male *Pls3*^fl^ mice; *N* = 3 of 48-wk-old female *Pls3*^fl/fl^;LysMCre^tg/0^ mice; *N* = 5 of 48-wk-old female *Pls3*^fl/fl^ mice; *N* = 5 of 48-wk-old male *Pls3*^fl^;LysMCre^tg/0^ mice; *N* = 4 of 48-wk-old male *Pls3*^fl^ mice. All results are shown as mean ± SEM. ^*^P < .05, ^*^^*^P < .01, ^*^^*^^*^P < .001, ns = not significant. An increase or decrease in comparison to the control group is indicated. Statistical test: Mann–Whitney U test.

**Table 5:** microCT data of the vertebrae T13 in 12-, 24-, and 48-wk-old female and male osteoclast-specific *Pls3* KO mice in comparison to their *Pls3*-floxed control littermates.

microCT of spine T13		12 wk	24 wk	48 wk
		*Pls3* ^fl/fl^;LysMCre vs *Pls3*^fl/fl^	*Pls3* ^fl/fl^;LysMCre vs *Pls3*^fl/fl^	*Pls3* ^fl/fl^;LysMCre vs *Pls3*^fl/fl^
BV/TV (%)	Female	ns	ns	ns
	Male	ns	ns	ns
BMD (mgHA/cm^3^)	Female	ns	ns	ns
	Male	ns	ns	ns
Tb.N (1/mm)	Female	ns	ns	ns
	Male	ns	ns	ns
Tb.Sp (mm)	Female	ns	ns	ns
	Male	ns	ns	ns
Tb.Th (mm)	Female	ns	ns	ns
	Male	ns	ns	ns
Conn.D (1/mm^3^)	Female	ns	ns	ns
	Male	ns	ns	ns
Vertebral foramen A/P (mm)	Female	ns	ns	ns
	Male	ns	ns	ns
Vertebral foramen M/L (mm)	Female	ns	ns	ns
	Male	^*^ increase	ns	ns
Cross-sectional area vertebral foramen (mm^2^)	Female	ns	ns	ns
	Male	ns	ns	ns
Vertebral body height (mm)	Female	ns	ns	ns
	Male	^*^ increase	ns	ns

Shown are bone volume fraction (BV/TV [%]),BMD (mgHA/cm^3^), trabecular number (Tb.N, 1/mm), trabecular separation (Tb.Sp, mm), trabecular thickness (Tb.Th, mm), and connectivity density (Conn.D, 1/mm^3^); vertebral foramen A/P (mm), vertebral foramen M/L (mm), cross-sectional area of the vertebral foramen (mm^2^), and vertebral body height (mm). *N* = 8 of 12-wk-old female *Pls3*^fl/fl^;LysMCre^tg/0^ mice; *N* = 12 of 12-wk-old female *Pls3*^fl/fl^ mice; *N* = 12 of 12-wk-old male *Pls3*^fl^;LysMCre^tg/0^ mice; *N* = 11 of 12-wk-old male *Pls3*^fl^ mice; *N* = 11 of 24-wk-old female *Pls3*^fl/fl^;LysMCre^tg/0^ mice; *N* = 13 of 24-wk-old female *Pls3*^fl/fl^ mice; *N* = 13 of 24-wk-old male *Pls3*^fl^;LysMCre^tg/0^ mice; *N* = 11 of 24-wk-old male *Pls3*^fl^ mice; *N* = 3 of 48-wk-old female *Pls3*^fl/fl^;LysMCre^tg/0^ mice; *N* = 5 of 48-wk-old female *Pls3*^fl/fl^ mice; *N* = 5 of 48-wk-old male *Pls3*^fl^;LysMCre^tg/0^ mice; *N* = 4 of 48-wk-old male *Pls3*^fl^ mice. All results are shown as mean ± SEM. ^*^P < .05, ^*^^*^P < .01, ^*^^*^^*^P < .001, ns = not significant. An increase or decrease in comparison to the control group is indicated. Statistical test: Mann–Whitney U test.

Furthermore, in 24-wk-old female osteoclast-specific *Pls3* KO mice, there was a significant increase in Tb.Th (6.5%, *P* < .05) and vertebral foramen A/P (3.7%, *P* < .05) measured for L4 (Supplemental Figure S5B, Table 2). In contrast, the remaining measured parameters of L4 and L5 were unaffected in 24-wk-old female and male osteoclast-specific *Pls3* KO mice (Supplemental Figures S5–S8, Tables 4 and 5). Likewise, no changes were detected for L4 and L5 of 48-wk-old mice (Supplemental Figures S5–S8C, Tables 2–5).

Similarly, most parameters of the thoracic regions T12 and T13 were unaffected in 24- and 48-wk-old female and male osteoclast-specific *Pls3* KO mice (Supplemental Figures S9–12B and C, Tables 4 and 5). Only the Conn.D (56.6%, *P* < .05) showed a significant increase in 48-wk-old female osteoclast-specific *Pls3* KO mice for T12 (Supplemental Figure S9C, Table 4).

### Absence of Pls3 in osteoclasts does not cause osteoporosis in the femur as measured by 3-PBT

**Figure 3:**
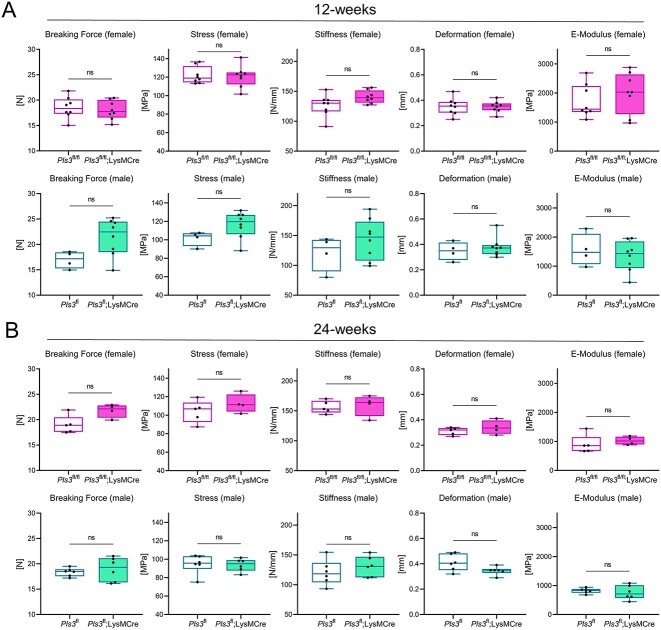
3-PBT of 12- and 24-wk-old female and male *Pls3*^fl/fl^;*LysM*Cre^tg/0^ mice in comparison to their *Pls3*^fl/fl^ littermates. (A) 12-wk-old and (B) 24-wk-old. Shown are breaking force (N), ultimate stress (MPa), stiffness (N/mm), deformation (mm), and elastic modulus (E-modulus, MPa). *N* = 8 of 12-wk-old female *Pls3*^fl/fl^;*LysM*Cre^tg/0^ mice; *N* = 8 of 12-wk-old female *Pls3*^fl/fl^ mice; *N* = 8 of 12-wk-old male *Pls3*^fl^;*LysM*Cre^tg/0^ mice; *N* = 4 of 12-wk-old male *Pls3*^fl^ mice; *N* = 4 of 24-wk-old female *Pls3*^fl/fl^;*LysM*Cre^tg/0^ mice; *N* = 5 of 24-wk-old female *Pls3*^fl/fl^ mice; *N* = 6 of 24-wk-old male *Pls3*^fl^;*LysM*Cre^tg/0^ mice; *N* = 6 of 24-wk-old male *Pls3*^fl^ mice. All results are shown as box plots, representing individual data points with median as a line, interquartile range (25th to 75th percentile), and min to max as whiskers. ^*^P < .05, ^*^^*^P < .01, ^*^^*^^*^P < .001, ns = not significant. Statistical test: Mann–Whitney U test.

To analyze the influence of PLS3 on the bone strength, 3-PBT of the femora in 12-wk-old female and male osteoclast-specific *Pls3* KO mice was performed and compared to sex-specific ubiquitous *Pls3* KO mice (Table 6).^20^ In osteoclast-specific *Pls3* KO females and males, all parameters were unaffected (Figure 3A, Supplemental Figure S13A, Table 6).^20^ In contrast, ubiquitous *Pls3* KO females and males showed a significant decrease in the ultimate stress. In addition, ubiquitous *Pls3* KO males showed reduced stiffness (Figure 3A, Table 6). Similar to the results in 12-wk-old osteoclast-specific *Pls3* KO mice, no changes were detected in 24- and 48-wk-old mice (Figure 3B, Supplemental Figure S13, Table 6). Overall, female and male osteoclast-specific *Pls3* KO mice did not exhibit an osteoporotic phenotype and did not resemble the phenotype of the ubiquitous *Pls3* KO mice.^20^

**Table 6:** 3-PBT data of the femurs in ubiquitous *Pls3* KO mice compared to osteoclast-specific *Pls3* KO mice.

3-PBT		12 wk		24 wk	48 wk
		*Pls3* KO vs WT	*Pls3* ^fl/fl^;LysMCre vs *Pls3*^fl/fl^	*Pls3* ^fl/fl^;LysMCre vs *Pls3*^fl/fl^	*Pls3* ^fl/fl^;LysMCre vs *Pls3*^fl/fl^
		Neugebauer et al.^20^	This study		
Breaking force (N)	Female	ns	ns	ns	ns
	Male	^*^^*^^*^ decrease	ns	ns	ns
Ultimate stress (MPa)	Female	^*^^*^ decrease	ns	ns	ns
	Male	^*^^*^^*^ decrease	ns	ns	ns
Stiffness (N/mm)	Female	ns	ns	ns	ns
	Male	^*^^*^^*^ decrease	ns	ns	ns
Deformation (mm)	Female	ns	ns	ns	ns
	Male	ns	ns	ns	ns
E-modulus (MPa)	Female	ns	ns	ns	ns
	Male	ns	ns	ns	ns

Previously, 3-PBT measurements were performed in 12-wk-old female and male ubiquitous *Pls3* KO mice and compared to WT mice.^20^ In this study, similar parameters were determined for femurs of 12-, 24-, and 48-wk-old female and male osteoclast-specific *Pls3* KO mice in comparison to their *Pls3*-floxed control littermates. Shown are breaking force (N), ultimate stress (MPa), stiffness (N/mm), deformation (mm), and elastic modulus (E-modulus, MPa). *N* = 10 of 12-wk-old *Pls3* KO mice; *N* = 10 of 12-wk-old WT mice; *N* = 8 of 12-wk-old female *Pls3*^fl/fl^;LysMCre^tg/0^ mice; *N* = 8 of 12-wk-old female *Pls3*^fl/fl^ mice; *N* = 8 of 12-wk-old male *Pls3*^fl^;LysMCre^tg/0^ mice; *N* = 4 of 12-wk-old male *Pls3*^fl^ mice; *N* = 4 of 24-wk-old female *Pls3*^fl/fl^;LysMCre^tg/0^ mice; *N* = 5 of 24-wk-old female *Pls3*^fl/fl^ mice; *N* = 6 of 24-wk-old male *Pls3*^fl^;LysMCre^tg/0^ mice; *N* = 6 of 24-wk-old male *Pls3*^fl^ mice; *N* = 5 of 48-wk-old female *Pls3*^fl/fl^;LysMCre^tg/0^ mice; *N* = 8 of 48-wk-old female *Pls3*^fl/fl^ mice; *N* = 8 of 48-wk-old male *Pls*3^fl^;LysMCre^tg/0^ mice; *N* = 11 of 48-wk-old male *Pls3*^fl^ mice. All results are shown as mean ± SEM. ^*^P < .05, ^*^^*^P < .01, ^*^^*^^*^P < .001, ns = not significant. An increase or decrease in comparison to the control group is indicated. Statistical test: Mann–Whitney U test.

## Discussion

To unravel the influence of PLS3 on bone morphology and osteoporosis, recently, a ubiquitous *Pls3* KO mouse model has been intensively studied with focus on bone morphometry.^20^ These findings suggest that ubiquitous *Pls3* KO mice, apart from decreased cortical and trabecular bone parameters and impaired strength and stiffness of bones, exhibit disturbed osteoclast formation, activity, and function with increased resorptive activity.^20^ However, these investigations did not allow the conclusion that osteoclasts are solely responsible for the observed osteoporotic phenotype, and thus, if mainly osteoclasts are affected in their functionality by aberrant PLS3 levels. In the present study, we aimed to unravel if, indeed, the osteoclasts are the main cell type affected in osteoporosis caused by the loss of PLS3. To address this question and to elucidate the role of PLS3 in osteoclast function and bone resorption during postnatal bone remodeling, we successfully generated an osteoclast-specific *Pls3* KO mouse model (Figure 1A). This allowed to investigate the isolated effect of *Pls3* loss in osteoclasts solely.

Differentiated osteoclasts from osteoclast-specific *Pls3* KO mice showed a strong resorptive capacity, which produced large resorptive areas when cultivated on bone slices. The resorptive efficiency was almost as strong as the activity of osteoclasts from ubiquitous *Pls3* KO mice (Figure 1C). These observations reflect an increased resorptive function caused by the loss of *Pls3* in osteoclasts, irrespective of the PLS3 level in other bone cells. Thus, we can conclude that the specific depletion of PLS3 in osteoclasts is sufficient to cause a hyperresorptive activity in vitro as is observed in osteoporosis in vivo.

Unexpectedly, the osteoclast-specific *Pls3* KO mice did not exhibit an osteoporotic phenotype as measured by microCT and 3-PBT at three different ages of the mice (12-, 24-, and 48-wk-old). Due to the strong resorptive activity of the osteoclasts, we expected a similar strong osteoporotic phenotype as observed in the ubiquitous *Pls3* KO mice.^20^ These contrasting outcomes may indicate that the observed osteoporotic phenotype in the ubiquitous *Pls3* KO mice was not solely caused by PLS3 level-dependent dysregulation of osteoclast function, but that other cell types of the bone were also affected by the loss of *Pls3*.^20^

Indeed, PLS3 is known to be expressed in various bone cell types, such as osteoblasts and osteocytes. In the osteoclast-specific *Pls3* KO mice, these cells might potentially compensate for the increased bone resorptive function of osteoclasts to maintain bone homeostasis.^18–21^ Importantly, the crosstalk and coupling between osteoblasts and osteoclasts regulate bone remodeling, increasing bone formation upon the increase of bone resorption.^35–38^ Thereby, the hyperactivity of osteoclasts in the osteoclast-specific *Pls3* KO might be balanced by the osteoblasts, preserving the bone architecture.

PLS3 is known to regulate the osteoblast mineralization and differentiation through its control of the osteoblasts’ Ca^2+^ concentrations.^13^^,^^18^^,^^39^ Although these crucial functions are unaffected in the osteoblasts of osteoclast-specific *Pls3* KO mice, it is likely that the ubiquitous *Pls3* KO might directly affect the osteoblasts’ function, being responsible for the contradictory results between the two mouse models. In fact, it was assumed previously that cortical thinning and osteoporosis development in human and mice with *PLS3* mutations or *Pls3* loss resulted from PLS3-dependent disturbances of the osteoblast-regulated bone mass acquisition and mineralization.^20^^,^^22^^,^^40^^,^^41^ Additionally, decreased osteoblast differentiation and bone formation, potentially caused by the impairment of Ca^2+^ regulation of PLS3 in osteoblasts, have been associated with *PLS3* mutations or *Pls3* loss, concomitantly increasing bone resorption.^13^^,^^40^^,^^42^ Likewise, mineralization defects and reduced osteoblast numbers were reported in human and rats with *PLS3* mutations, leading to osteoporosis.^11^^,^^41^^,^^43–45^ Based on these studies, and due to the absence of an osteoporotic phenotype in the osteoclast-specific *Pls3* KO mice, it is very likely that the ubiquitous *Pls3* KO equally affects the function of osteoblasts and osteoclasts, therefore interfering with the balance between osteoblast-mediated bone formation and osteoclast-mediated bone resorption, where bone resorption exceeds bone formation.

Likewise, PLS3, through its function in cytoskeleton modification and suggested involvement in dendrite formation, is assumed to be indispensable for osteocytes’ activity and function, thereby regulating the sensing of mechanical stimuli and mechanotransduction of osteocytes. In this way, PLS3 is involved in the regulation of communication and networking between osteocytes, osteoblasts, and osteoclasts, and subsequently controls bone homeostasis.^11^^,^^46–49^ Hence, osteocytes of the osteoclast-specific *Pls3* KO mice could be able to respond to the abnormal increased osteoclast function by regulating the osteoblast bone formation. However, this regulatory function might be disturbed upon ubiquitous *Pls3* KO, rendering osteocytes incapable to compensate for the increased bone resorptive function of osteoclasts in these mice. In line with this, *PLS3* mutations showed to alter osteocyte mechanotransduction, causing the misbalancing of bone homeostasis.^11^^,^^44^ Further studies on *PLS3* mutations reported abnormal osteoid accumulations in focal and perilacunar areas of osteocytes, accompanied by an increased osteocyte apoptosis rate, potentially resulting in osteoclast recruitment and osteoclastogenesis to initiate bone resorption.^50–52^ Thus, dysregulations of the *PLS3* level in osteocytes could even augment the effect of the increased osteoclast bone resorption in the ubiquitous *Pls3* KO mice, uncoupling bone resorption from bone formation, which contributes loss of trabecular and cortical bone.^53–55^

Thus, to identify the cell system, which is mostly affected by the loss of *Pls3*, we believe it is crucial to deeply investigate alternative cell types of the bone, especially osteoblasts and osteocytes, as well as to study the crosstalk between osteoblasts and osteoclasts in the ubiquitous *Pls3* KO mice. In this regard, the generation of combinatorial cell–specific *Pls3* KO mouse lines will be needed to understand the dependence of specific cells on PLS3 and unveil the pathomechanisms involved.

There are limitations to consider in the present study. No dynamic histomorphometric changes could be analyzed since calcein labeling was not performed to determine the rate of bone formation and mineralization. Additionally, in vivo measurements of the resorptive activity of osteoclasts from osteoclast-specific *Pls3* KO mice are lacking in the present study. Thus, in future studies, the coupling between osteoclasts and osteoblasts should further be investigated by in vivo methods such as CTX-1 ELISA to reveal if alternative cell types of the bone, such as osteoblasts are involved in the bone phenotype.

In conclusion, we confirm that PLS3 influences the osteoclast activity by increasing their resorptive function. However, the specific loss of *Pls3* in osteoclasts is not sufficient to cause the osteoporotic phenotype in vivo as observed in the ubiquitous *Pls3* KO mice.^20^

Moreover, our results highlight the strong dependence of the whole organism and specifically bone cells on regulated *PLS3* levels. We conclude that the development of the PLS3-associated osteoporotic phenotype is highly complex and cannot be reproduced in a system singularly focused on one cell type.

In addition, we were able to develop a novel polyclonal PLS3 antibody, which specifically visualized filamentous structures at the cell edge, colocalizing with F-actin, in WT control osteoclasts and MEFs (Figure 1B, Supplemental Figure 2C). This specific localization of PLS3 to F-actin rich structures at the cell edge, focal adhesions, and stress fibers is in line with previous findings in HEK293 cells, fibroblasts, and U2OS osteoblasts, confirming the specificity of the antibody.^13^^,^^56^

## Supplementary Material

SuppInfoPlus_Legends_ziad009

## Data Availability

The data that support the findings of this study are available from the corresponding authors upon reasonable request.
