# Correction to: Osteoclast-specific Plastin 3 knockout in mice fail to develop osteoporosis despite dramatic increased osteoclast resorption activity

**DOI:** 10.1093/jbmrpl/ziae042

**Published:** 2024-03-27

This is a correction to: Ilka Maus, Maren Dreiner, Sebastian Zetzsche, Fabian Metzen, Bryony C Ross, Daniela Mählich, Manuel Koch, Anja Niehoff, Brunhilde Wirth, Osteoclast-specific Plastin 3 knockout in mice fail to develop osteoporosis despite dramatic increased osteoclast resorption activity, *JBMR Plus*, Volume 8, Issue 1, January 2024, ziad009, https://doi.org/10.1093/jbmrpl/ziad009

Following the original publication of this article, a reader queried the axis unit details of Figure 1C and the statistical comparisons shown. The authors have now revised the figure and its accompanying statistical analysis in the updated figure and legend. The Methods have also been updated to reflect this change, and the statistician consulted for this analysis is now recognized in updated Acknowledgments section. All co-authors have approved these changes.

