# Supplementary material for: Osteoclast-specific Plastin 3 knockout in mice fail to develop osteoporosis despite dramatic increased osteoclast resorption activity
Source: JBMR Plus. 2024 Jan 4;8(1):ziad009. doi: 10.1093/jbmrpl/ziad009 (PMC10971598; doi:10.1093/jbmrpl/ziad009)
Supplement: SuppInfoPlus_Legends_ziad009 [file suppinfoplus_legends_ziad009.pdf]

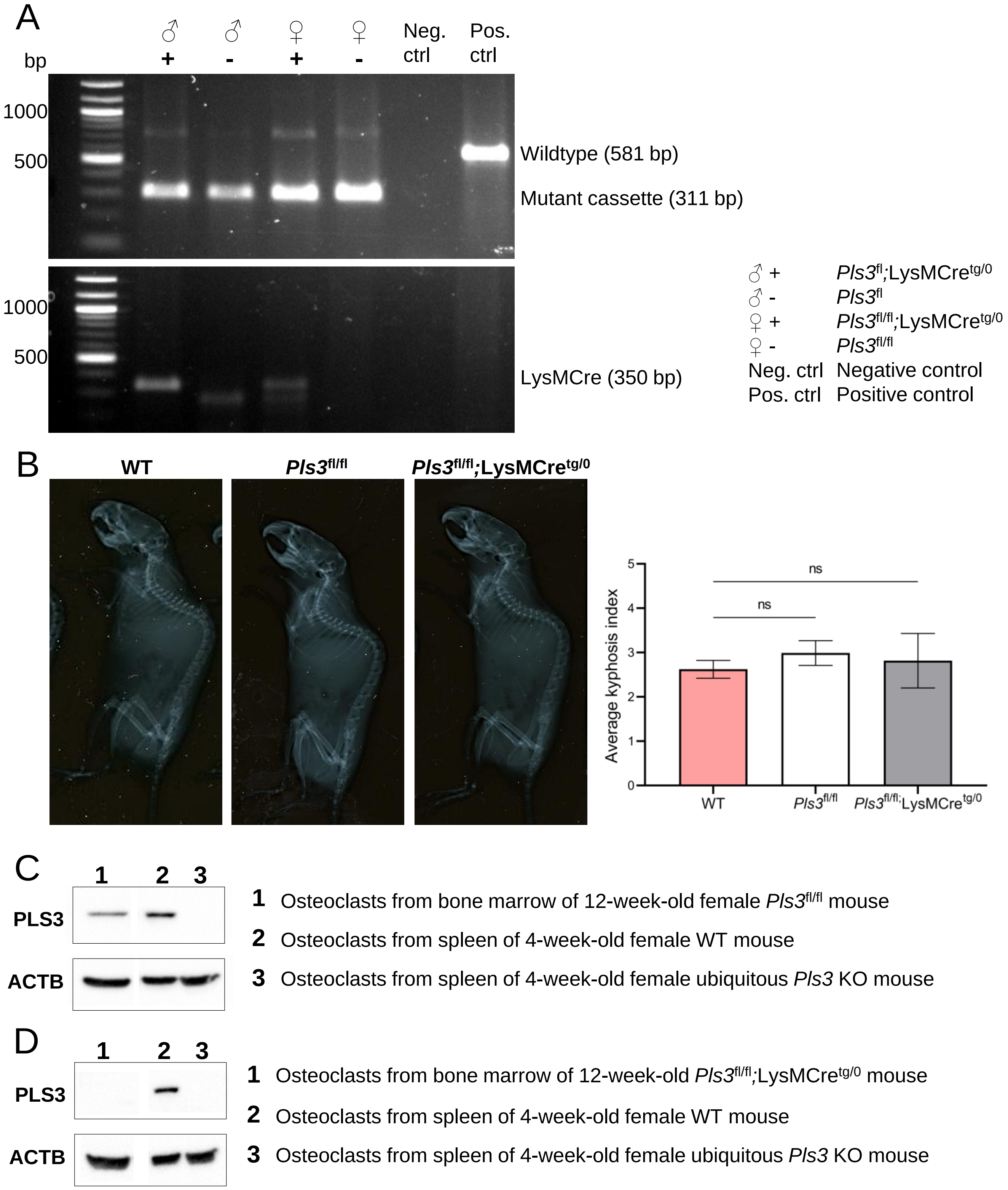

Supplemental Fig. S1: Generation and characterization of osteoclast-specific *Pls3* KO (*Pls3*$^{fl/fl}$;LysMCre$^{tg/0}$) mice. (A) Genotyping of male and female osteoclast-specific *Pls3* KO mice and *Pls3*-floxed mice confirmed the successful removal of the FRT sites resulting in floxed *Pls3* alleles (cassette) (upper parts), and the presence of LysMCre (lower part). A WT sample was used as positive control. (B) Exemplary images of the lateral view of mouse whole body radiographs of 12-week-old female WT, *Pls3*$^{fl/fl}$, and *Pls3*$^{fl/fl}$;LysMCre$^{tg/0}$ mice used for the calculation of the kyphosis index. For the kyphosis index measurements and calculations, female and male osteoclast-specific *Pls3* KO mice and *Pls3*-floxed mice were used. (C) PLS3 levels in osteoclasts derived from bone marrow of 12-week-old *Pls3*$^{fl/fl}$ and (D) *Pls3*$^{fl/fl}$;LysMCre$^{tg/0}$ mouse (lane 1) and from osteoclasts derived from spleen of 4-week-old WT and ubiquitous *Pls3* KO mouse (lane 2 and 3). Exposure 55kV/30seconds. N = 6-10. All results are shown as mean ± SD. **P* < 0.05, ***P* < 0.01, ****P* < 0.001, ns = not significant. Statistical test: unpaired two-tailed Student's *t*-test.

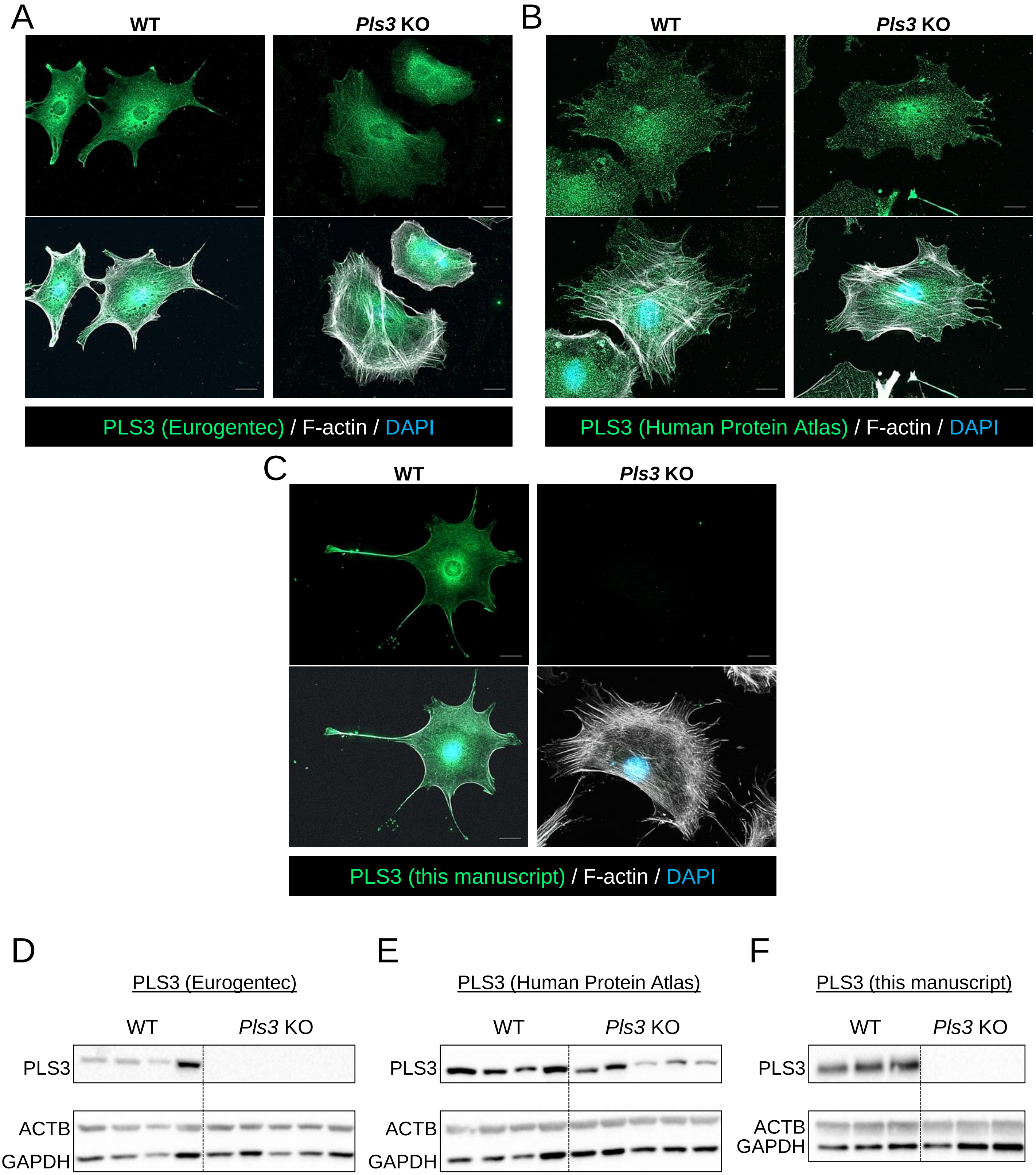

Supplemental Fig. S2: Determination of the specificity of different PLS3 antibodies on cultivated murine embryonic fibroblasts (MEFs) and osteoclasts from WT and *Pls3* KO mice. (A-C) Immunofluorescence staining of cultivated WT control and *Pls3* KO MEFs using (A) PLS3 antibody from Eurogentec (1238), (B) PLS3 antibody from Human Protein Atlas (HPA020433), and (C) PLS3 antibody developed in this manuscript (3772). PLS3 is shown in green, F-actin in white, and nuclei are stained in blue. (D-F) Western blot analysis of PLS3 using proteins from WT control and *Pls3* KO primary differentiated osteoclasts with (A) PLS3 antibody from Eurogentec (1238), (B) PLS3 antibody from Human Protein Atlas (HPA020433), and (C) PLS3 antibody developed in this manuscript (3772). Scale bar: 20 µm. (D-F) N=3-5.

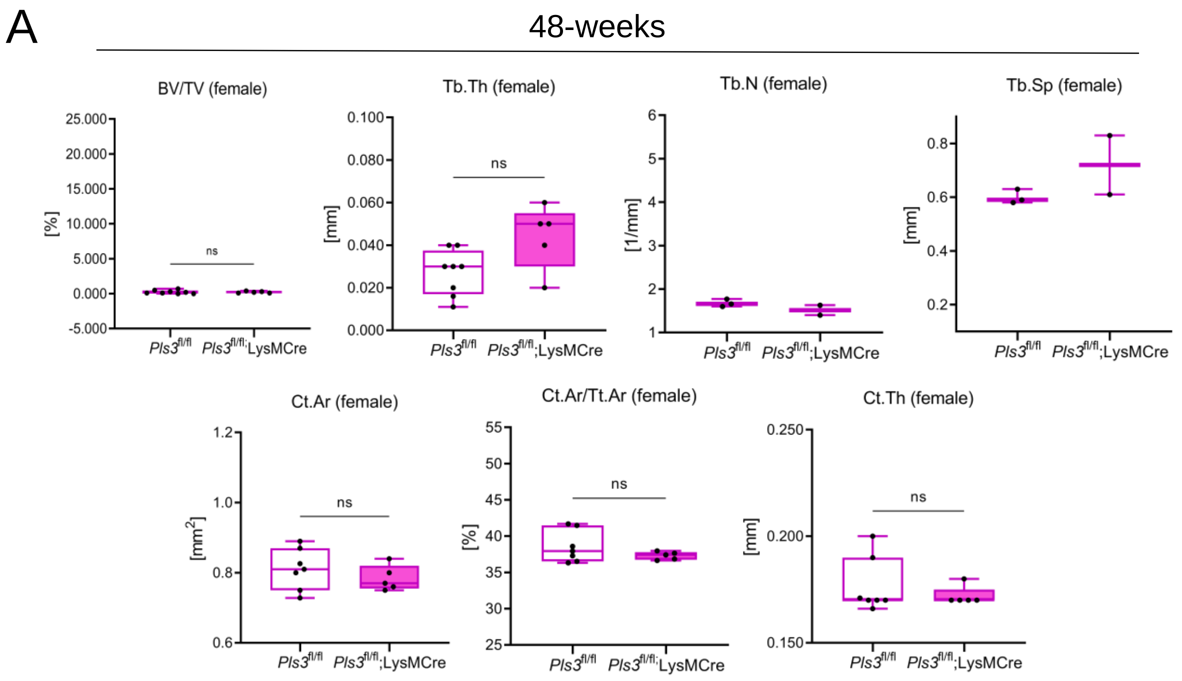

Supplemental Fig. S3: MicroCT data of the femora from 48-week-old female *Pls3*$^{fl/fl}$;LysMCre$^{tg/0}$ mice and their *Pls3*$^{fl/fl}$ littermates. . Shown are bone volume fraction (BV/TV, %), trabecular thickness (Tb.Th, mm), trabecular number (Tb.N, 1/mm), trabecular separation (Tb.Sp, mm), cortical area (Ct.Ar, mm²), ratio of Ct.Area to tissue area (Ct.Ar/Tt.Ar, %) ratio and cortical thickness (Ct.Th, mm). N = 2-5 of 48-week-old female *Pls3*$^{fl/fl}$;LysMCre$^{tg/0}$ mice; N = 3-8 of 48-week-old female *Pls3*$^{fl/fl}$ mice. All results are shown as box plots, representing individual data points with median as a line, interquartile range (25th to 75th percentile), and min to max as whiskers. *$P < 0.05$, **$P < 0.01$, ***$P < 0.001$, ns = not significant. Statistical test: Mann-Whitney U test.

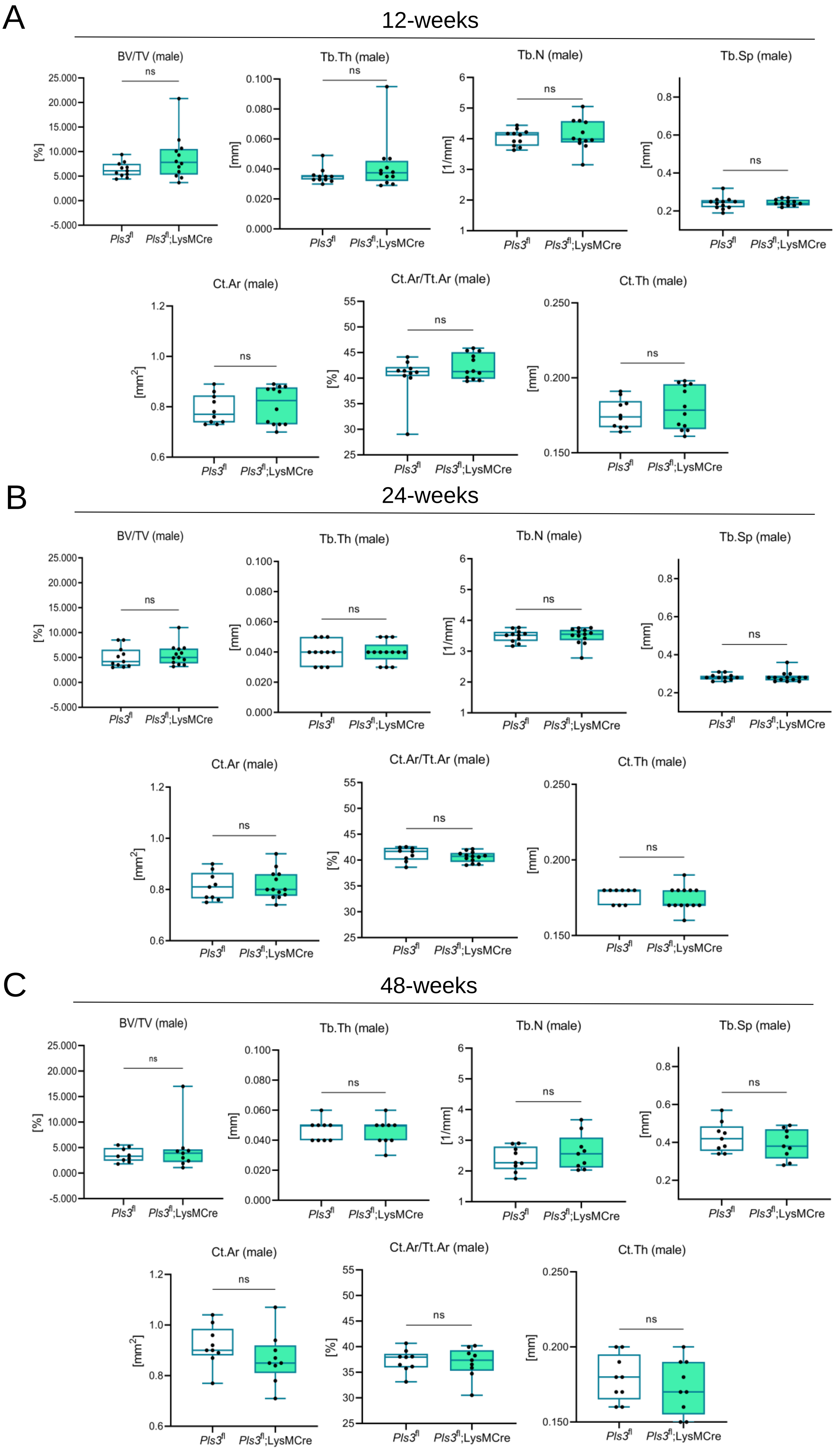

A
12-weeks
BV/TV (male)
Tb.Th (male)
Tb.N (male)
Tb.Sp (male)
Ct.Ar (male)
Ct.Ar/Tt.Ar (male)
Ct.Th (male)
B
24-weeks
BV/TV (male)
Tb.Th (male)
Tb.N (male)
Tb.Sp (male)
Ct.Ar (male)
Ct.Ar/Tt.Ar (male)
Ct.Th (male)
C
48-weeks
BV/TV (male)
Tb.Th (male)
Tb.N (male)
Tb.Sp (male)
Ct.Ar (male)
Ct.Ar/Tt.Ar (male)
Ct.Th (male)
ns
[%]
[mm]
[1/mm]
[mm2]
Pls3fl
Pls3fl;LysMCre

Supplemental Fig. S4: MicroCT data of femora from male *Pls3*^{fl};LysMCre$^{tg/0}$ mice and their *Pls3*^{fl} littermates. Shown are bone volume fraction (BV/TV, %), bone mineral density (BMD, mmHA/cm^3), trabecular number (Tb.N, 1/mm), trabecular separation (Tb.Sp, mm), trabecular thickness (Tb.Th, mm), and connectivity density (Conn.D, 1/mm^3), vertebral foramen A/P (mm), vertebral foramen M/L (mm), cross sectional area of the vertebral foramen (mm^2) and vertebral body height (mm) for (A) 12-, (B) 24-, and (C) 48-week-old mice. N = 12 of 12-week-old male *Pls3*^{fl};LysMCre$^{tg/0}$ mice; N = 11 of 12-week-old male *Pls3*^{fl} mice; N = 13 of 24-week-old male *Pls3*^{fl};LysMCre$^{tg/0}$ mice; N = 9-11 of 24-week-old male *Pls3*^{fl} mice; N = 9 of 48-week-old male *Pls3*^{fl};LysMCre$^{tg/0}$ mice; N = 9 of 48-week-old male *Pls3*^{fl} mice. All results are shown as box plots, representing individual data points with median as a line, interquartile range (25th to 75th percentile), and min to max as whiskers. *$P < 0.05$, **$P < 0.01$, ***$P < 0.001$, ns = not significant. Statistical test: Mann-Whitney U test.

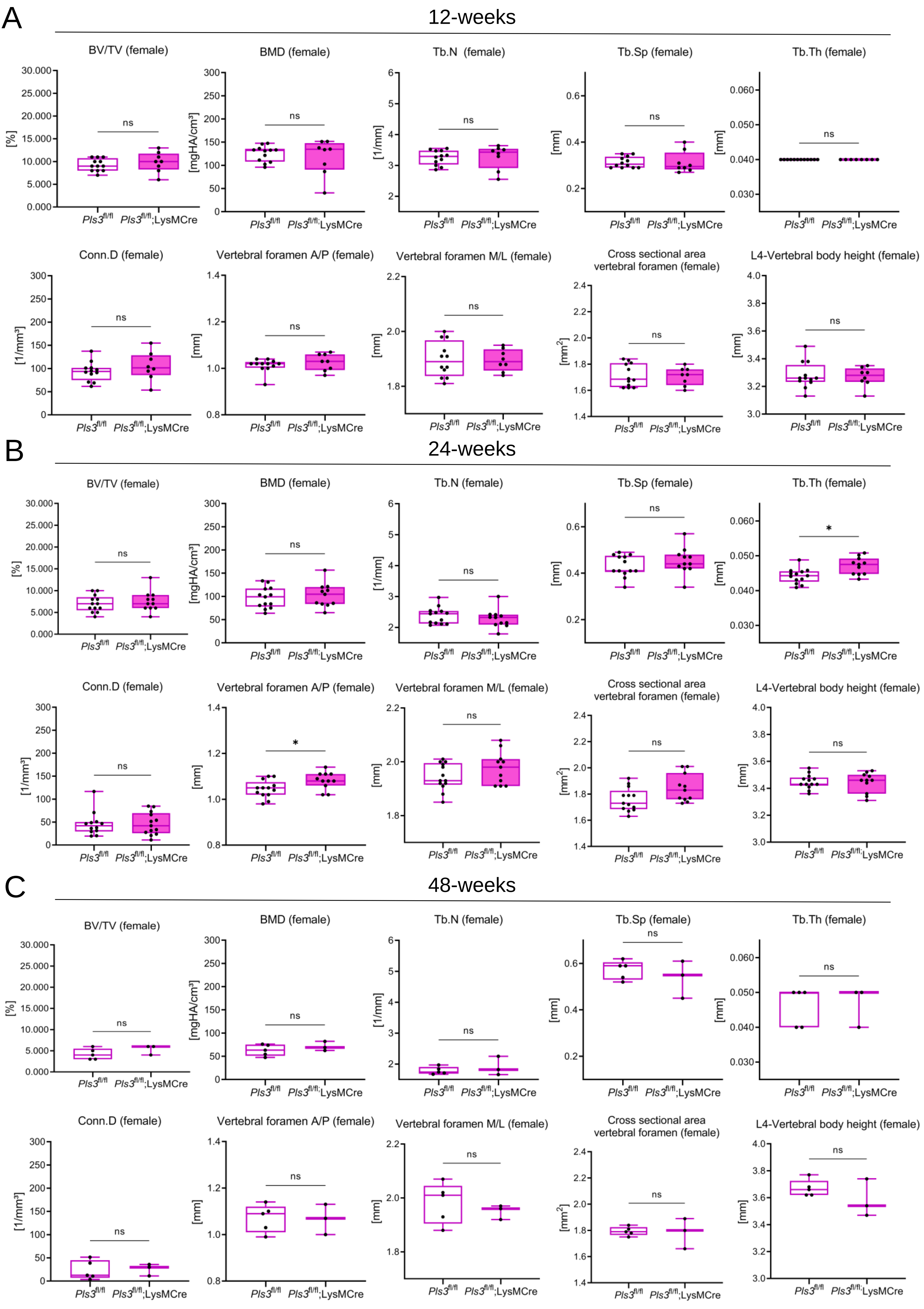

A
12-weeks
BV/TV (female)
BMD (female)
Tb.N (female)
Tb.Sp (female)
Tb.Th (female)
Conn.D (female)
Vertebral foramen A/P (female)
Vertebral foramen M/L (female)
Cross sectional area vertebral foramen (female)
L4-Vertebral body height (female)
B
24-weeks
C
48-weeks
ns
*
[%]
[mgHA/cm³]
[1/mm]
[mm]
[1/mm³]
[mm²]
Pls3fl/fl
Pls3fl/fl;LysMCre

Supplemental Fig. S5: MicroCT data of spine L4 from female *Pls3*$^{fl/fl}$;LysMCre$^{tg/0}$ mice in comparison to their *Pls3*$^{fl/fl}$ littermates. Shown are bone volume fraction (BV/TV, %), bone mineral density (BMD, mmHA/cm^3), trabecular number (Tb.N, 1/mm), trabecular separation (Tb.Sp, mm), trabecular thickness (Tb.Th, mm), and connectivity density (Conn.D, 1/mm^3), vertebral foramen A/P (mm), vertebral foramen M/L (mm), cross sectional area of the vertebral foramen (mm^2) and vertebral body height (mm) for (A) 12-, (B) for 24-, and (C) 48-week-old female mice. N = 8 of 12-week-old female *Pls3*$^{fl/fl}$;LysMCre$^{tg/0}$ mice; N = 12 of 12-week-old female *Pls3*$^{fl/fl}$ mice; N = 11 of 24-week-old female *Pls3*$^{fl/fl}$;LysMCre$^{tg/0}$ mice; N = 13 of 24-week-old female *Pls3*$^{fl/fl}$ mice; N = 3 of 48-week-old female *Pls3*$^{fl/fl}$;LysMCre$^{tg/0}$ mice; N = 5 of 48-week-old female *Pls3*$^{fl/fl}$ mice. All results are shown as box plots, representing individual data points with median as a line, interquartile range (25th to 75th percentile), and min to max as whiskers. $^*P < 0.05$, $^{**}P < 0.01$, $^{***}P < 0.001$, ns = not significant. Statistical test: Mann-Whitney U test.

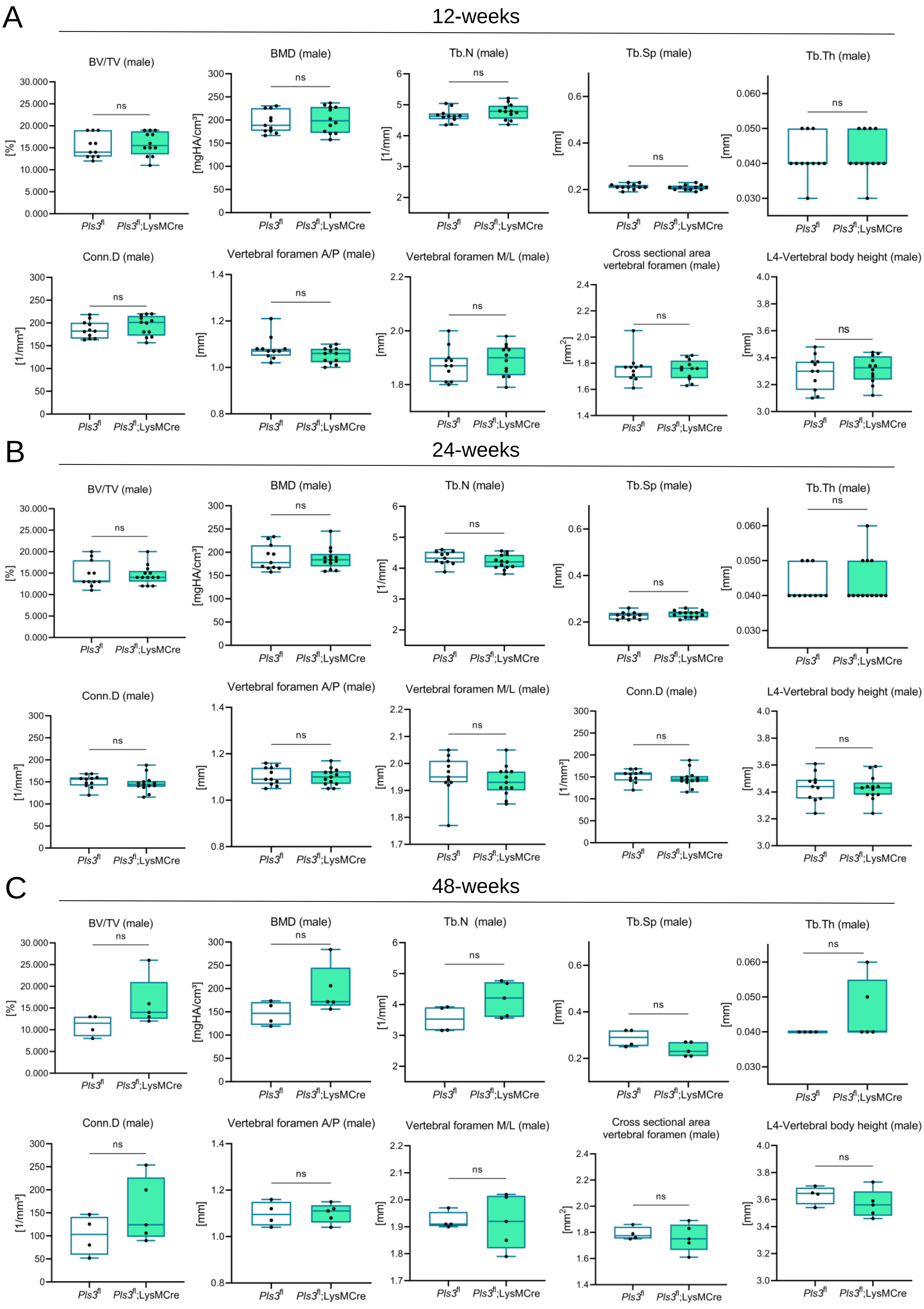
A
12-weeks
BV/TV (male) [%]
BMD (male) [mgHA/cm³]
Tb.N (male) [1/mm]
Tb.Sp (male) [mm]
Tb.Th (male) [mm]
Conn.D (male) [1/mm³]
Vertebral foramen A/P (male) [mm]
Vertebral foramen M/L (male) [mm]
Cross sectional area vertebral foramen (male) [mm²]
L4-Vertebral body height (male) [mm]
B
24-weeks
BV/TV (male) [%]
BMD (male) [mgHA/cm³]
Tb.N (male) [1/mm]
Tb.Sp (male) [mm]
Tb.Th (male) [mm]
Conn.D (male) [1/mm³]
Vertebral foramen A/P (male) [mm]
Vertebral foramen M/L (male) [mm]
Conn.D (male) [1/mm³]
L4-Vertebral body height (male) [mm]
C
48-weeks
BV/TV (male) [%]
BMD (male) [mgHA/cm³]
Tb.N (male) [1/mm]
Tb.Sp (male) [mm]
Tb.Th (male) [mm]
Conn.D (male) [1/mm³]
Vertebral foramen A/P (male) [mm]
Vertebral foramen M/L (male) [mm]
Cross sectional area vertebral foramen (male) [mm²]
L4-Vertebral body height (male) [mm]
ns
Pls3fl
Pls3fl;LysMCre

Supplemental Fig. S6: MicroCT data of spine L4 from male *Pls3*^{fl};LysMCre$^{tg/0}$ mice in comparison to their *Pls3*^{fl} littermates. Shown are bone volume fraction (BV/TV, %), bone mineral density (BMD, mmHA/cm^3), trabecular number (Tb.N, 1/mm), trabecular separation (Tb.Sp, mm), trabecular thickness (Tb.Th, mm), and connectivity density (Conn.D, 1/mm^3), vertebral foramen A/P (mm), vertebral foramen M/L (mm), cross sectional area of the vertebral foramen (mm^2) and vertebral body height (mm) for (A) 12-, (B) for 24-, and (C) 48-week-old male mice. N = 12 of 12-week-old male *Pls3*^{fl};LysMCre$^{tg/0}$ mice; N = 11 of 12-week-old male *Pls3*^{fl} mice; N =13 of 24-week-old male *Pls3*^{fl};LysMCre$^{tg/0}$ mice; N = 11 of 24-week-old male *Pls3*^{fl} mice; N = 5 of 48-week-old male *Pls3*^{fl};LysMCre$^{tg/0}$ mice; N = 4 of 48-week-old male *Pls3*^{fl} mice. All results are shown as box plots, representing individual data points with median as a line, interquartile range (25th to 75th percentile), and min to max as whiskers. *P < 0.05, **P < 0.01, ***P < 0.001, ns = not significant. Statistical test: Mann-Whitney U test.

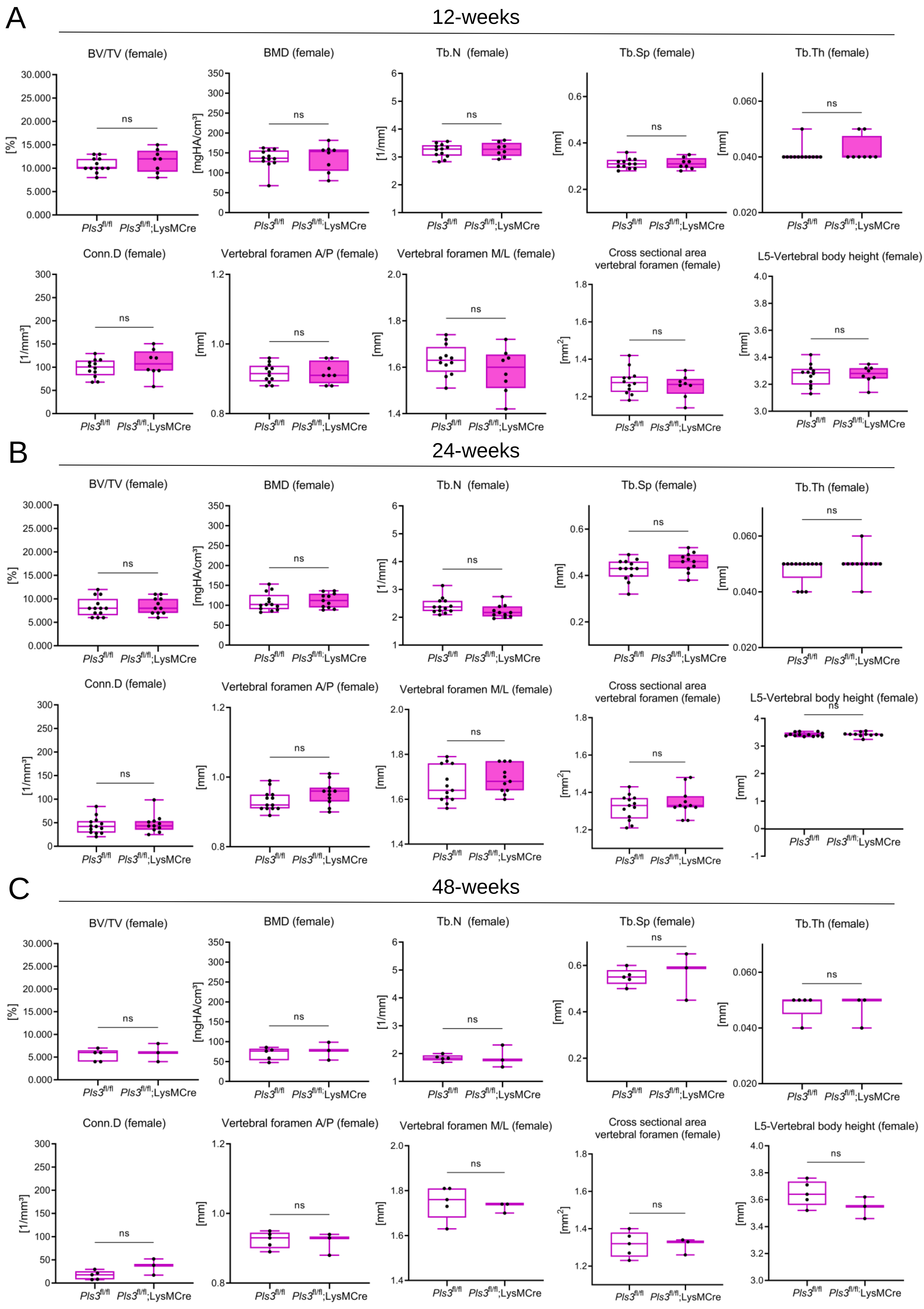
A
12-weeks
B
24-weeks
C
48-weeks
BV/TV (female)
BMD (female)
Tb.N (female)
Tb.Sp (female)
Tb.Th (female)
Conn.D (female)
Vertebral foramen A/P (female)
Vertebral foramen M/L (female)
Cross sectional area vertebral foramen (female)
L5-Vertebral body height (female)
[%]
[mgHA/cm³]
[1/mm]
[mm]
[1/mm³]
[mm²]
ns
Pls3fl/fl
Pls3fl/fl;LysMCre

Supplemental Fig. S7: MicroCT data of spine L5 from female *Pls3*$^{fl/fl}$;LysMCre$^{tg/0}$ mice in comparison to their *Pls3*$^{fl/fl}$ littermates. Shown are bone volume fraction (BV/TV, %), bone mineral density (BMD, mmHA/cm^3), trabecular number (Tb.N, 1/mm), trabecular separation (Tb.Sp, mm), trabecular thickness (Tb.Th, mm), and connectivity density (Conn.D, 1/mm^3), vertebral foramen A/P (mm), vertebral foramen M/L (mm), cross sectional area of the vertebral foramen (mm^2) and vertebral body height (mm) for (A) 12-, (B) for 24-, and (C) 48-week-old female mice. N = 8 of 12-week-old female *Pls3*$^{fl/fl}$;LysMCre$^{tg/0}$ mice; N = 12 of 12-week-old female *Pls3*$^{fl/fl}$ mice; N = 11 of 24-week-old female *Pls3*$^{fl/fl}$;LysMCre$^{tg/0}$ mice; N = 13 of 24-week-old female *Pls3*$^{fl/fl}$ mice; N = 3 of 48-week-old female *Pls3*$^{fl/fl}$;LysMCre$^{tg/0}$ mice; N = 5 of 48-week-old female *Pls3*$^{fl/fl}$ mice. All results are shown as box plots, representing individual data points with median as a line, interquartile range (25th to 75th percentile), and min to max as whiskers. *$P < 0.05$, **$P < 0.01$, ***$P < 0.001$, ns = not significant. Statistical test: Mann-Whitney U test.

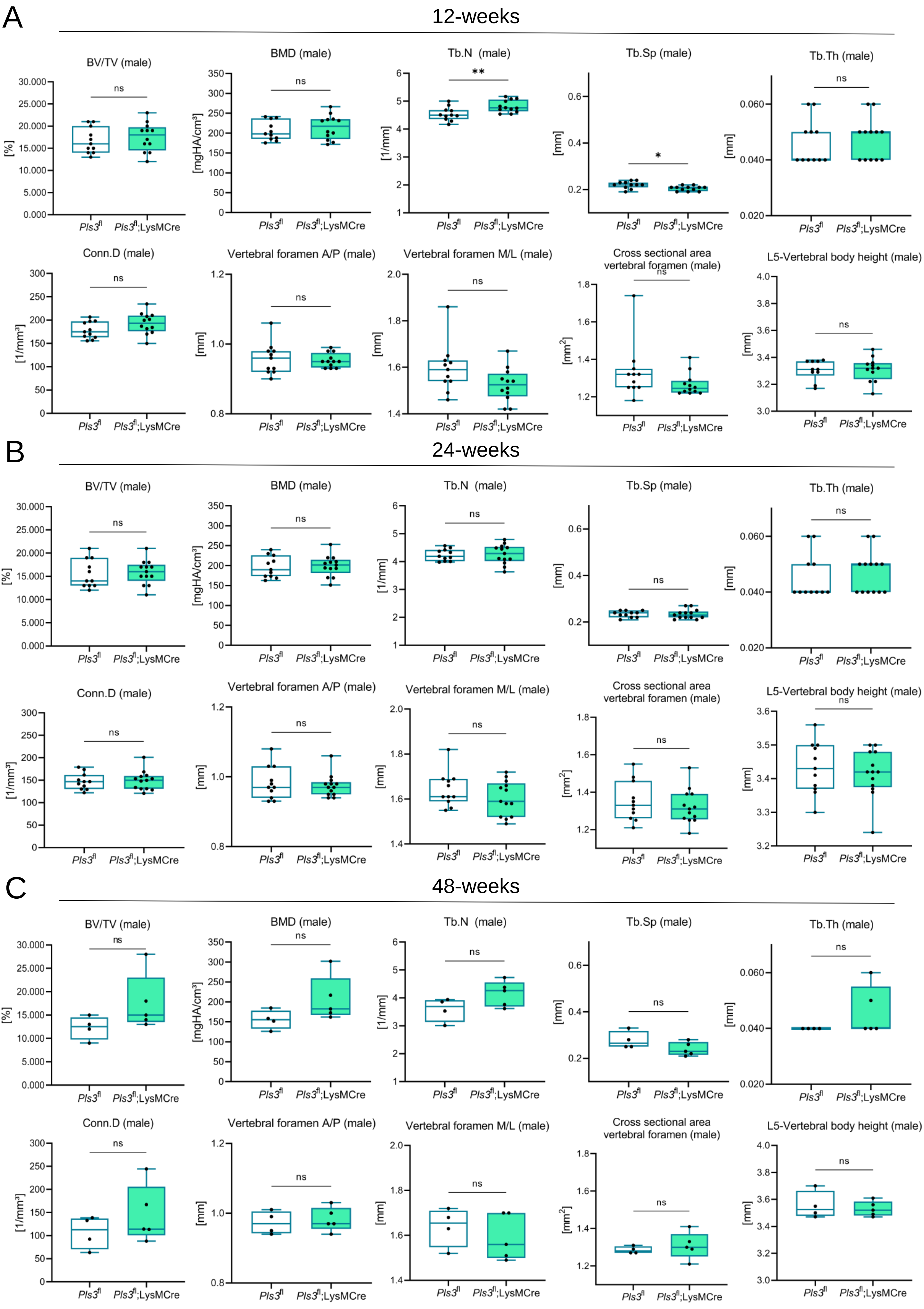

A
12-weeks
BV/TV (male)
BMD (male)
Tb.N (male)
Tb.Sp (male)
Tb.Th (male)
Conn.D (male)
Vertebral foramen A/P (male)
Vertebral foramen M/L (male)
Cross sectional area vertebral foramen (male)
L5-Vertebral body height (male)
ns
**
*
[%]
[mgHA/cm³]
[1/mm]
[mm]
[1/mm³]
[mm²]
Pls3fl
Pls3fl;LysMCre
B
24-weeks
C
48-weeks

Supplemental Fig. S8: MicroCT data of spine L5 from male *Pls3*^{fl};LysMCre$^{tg/0}$ mice in comparison to their *Pls3*^{fl} littermates. Shown are bone volume fraction (BV/TV, %), bone mineral density (BMD, mmHA/cm^3), trabecular number (Tb.N, 1/mm), trabecular separation (Tb.Sp, mm), trabecular thickness (Tb.Th, mm), and connectivity density (Conn.D, 1/mm^3), vertebral foramen A/P (mm), vertebral foramen M/L (mm), cross sectional area of the vertebral foramen (mm^2) and vertebral body height (mm) for (A) 12-, (B) for 24-, and (C) 48-week-old male mice. N = 12 of 12-week-old male *Pls3*^{fl};LysMCre$^{tg/0}$ mice; N = 11 of 12-week-old male *Pls3*^{fl} mice; N = 13 of 24-week-old male *Pls3*^{fl};LysMCre$^{tg/0}$ mice; N = 11 of 24-week-old male *Pls3*^{fl} mice; N = 5 of 48-week-old male *Pls3*^{fl};LysMCre$^{tg/0}$ mice; N = 4 of 48-week-old male *Pls3*^{fl} mice. All results are shown as box plots, representing individual data points with median as a line, interquartile range (25th to 75th percentile), and min to max as whiskers. *P < 0.05, **P < 0.01, ***P < 0.001, ns = not significant. Statistical test: Mann-Whitney U test.

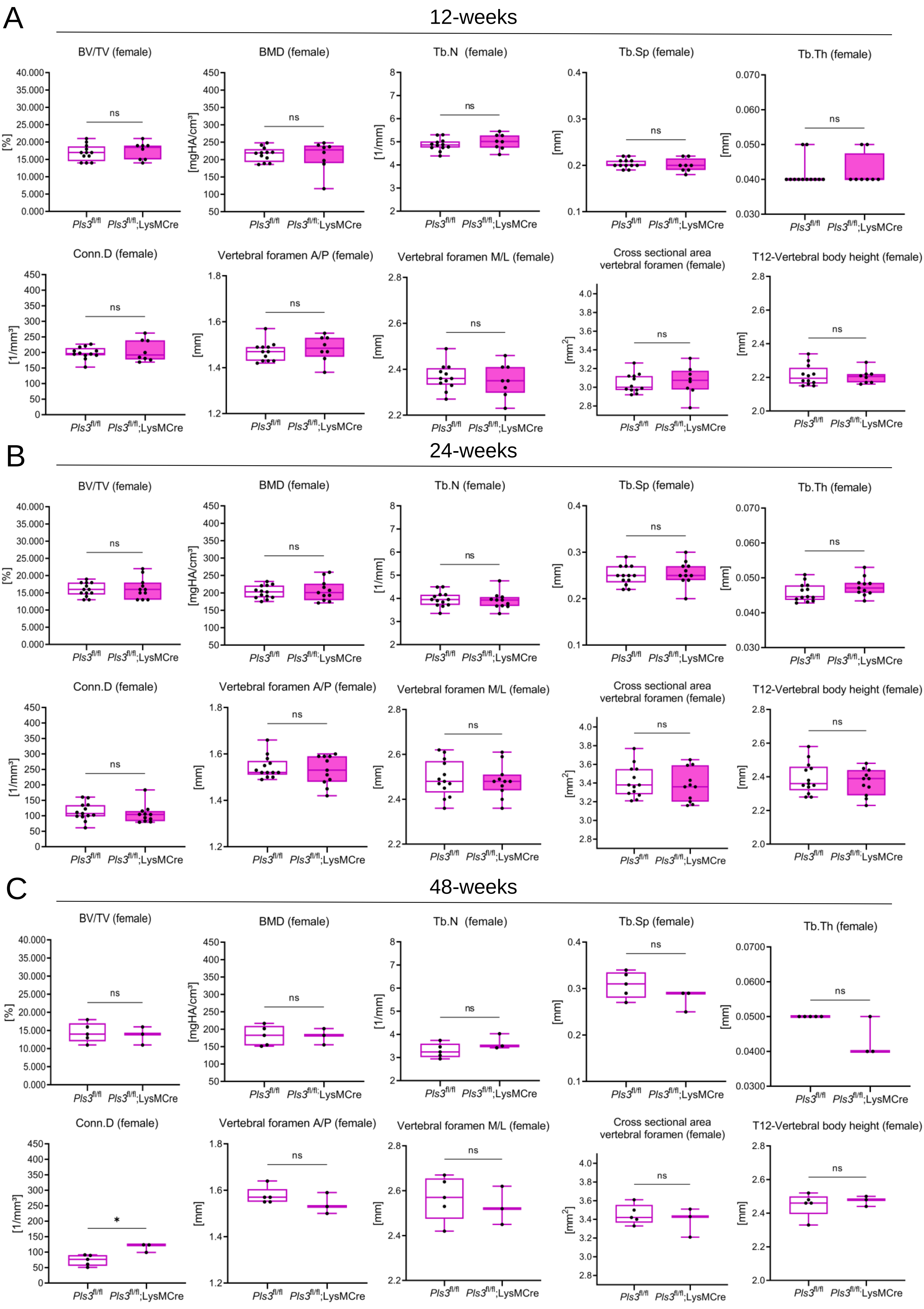
A
12-weeks
BV/TV (female)
BMD (female)
Tb.N (female)
Tb.Sp (female)
Tb.Th (female)
Conn.D (female)
Vertebral foramen A/P (female)
Vertebral foramen M/L (female)
Cross sectional area vertebral foramen (female)
T12-Vertebral body height (female)
B
24-weeks
BV/TV (female)
BMD (female)
Tb.N (female)
Tb.Sp (female)
Tb.Th (female)
Conn.D (female)
Vertebral foramen A/P (female)
Vertebral foramen M/L (female)
Cross sectional area vertebral foramen (female)
T12-Vertebral body height (female)
C
48-weeks
BV/TV (female)
BMD (female)
Tb.N (female)
Tb.Sp (female)
Tb.Th (female)
Conn.D (female)
Vertebral foramen A/P (female)
Vertebral foramen M/L (female)
Cross sectional area vertebral foramen (female)
T12-Vertebral body height (female)
[%]
[mgHA/cm³]
[1/mm]
[mm]
[1/mm³]
[mm²]
ns
*
Pls3fl/fl
Pls3fl/fl;LysMCre

Supplemental Fig. S9: MicroCT data of spine T12 from female *Pls3*$^{fl/fl}$;LysMCre$^{tg/0}$ mice in comparison to their *Pls3*$^{fl/fl}$ littermates. Shown are bone volume fraction (BV/TV, %), bone mineral density (BMD, mmHA/cm^3), trabecular number (Tb.N, 1/mm), trabecular separation (Tb.Sp, mm), trabecular thickness (Tb.Th, mm), and connectivity density (Conn.D, 1/mm^3), vertebral foramen A/P (mm), vertebral foramen M/L (mm), cross sectional area of the vertebral foramen (mm^2) and vertebral body height (mm) for (A) 12-, (B) for 24-, and (C) 48-week-old female mice. N = 8 of 12-week-old female *Pls3*$^{fl/fl}$;LysMCre$^{tg/0}$ mice; N = 12 of 12-week-old female *Pls3*$^{fl/fl}$ mice; N = 11 of 24-week-old female *Pls3*$^{fl/fl}$;LysMCre$^{tg/0}$ mice; N = 13 of 24-week-old female *Pls3*$^{fl/fl}$ mice; N = 3 of 48-week-old female *Pls3*$^{fl/fl}$;LysMCre$^{tg/0}$ mice; N = 5 of 48-week-old female *Pls3*$^{fl/fl}$ mice. All results are shown as box plots, representing individual data points with median as a line, interquartile range (25th to 75th percentile), and min to max as whiskers. *$P < 0.05$, **$P < 0.01$, ***$P < 0.001$, ns = not significant. Statistical test: Mann-Whitney U test.

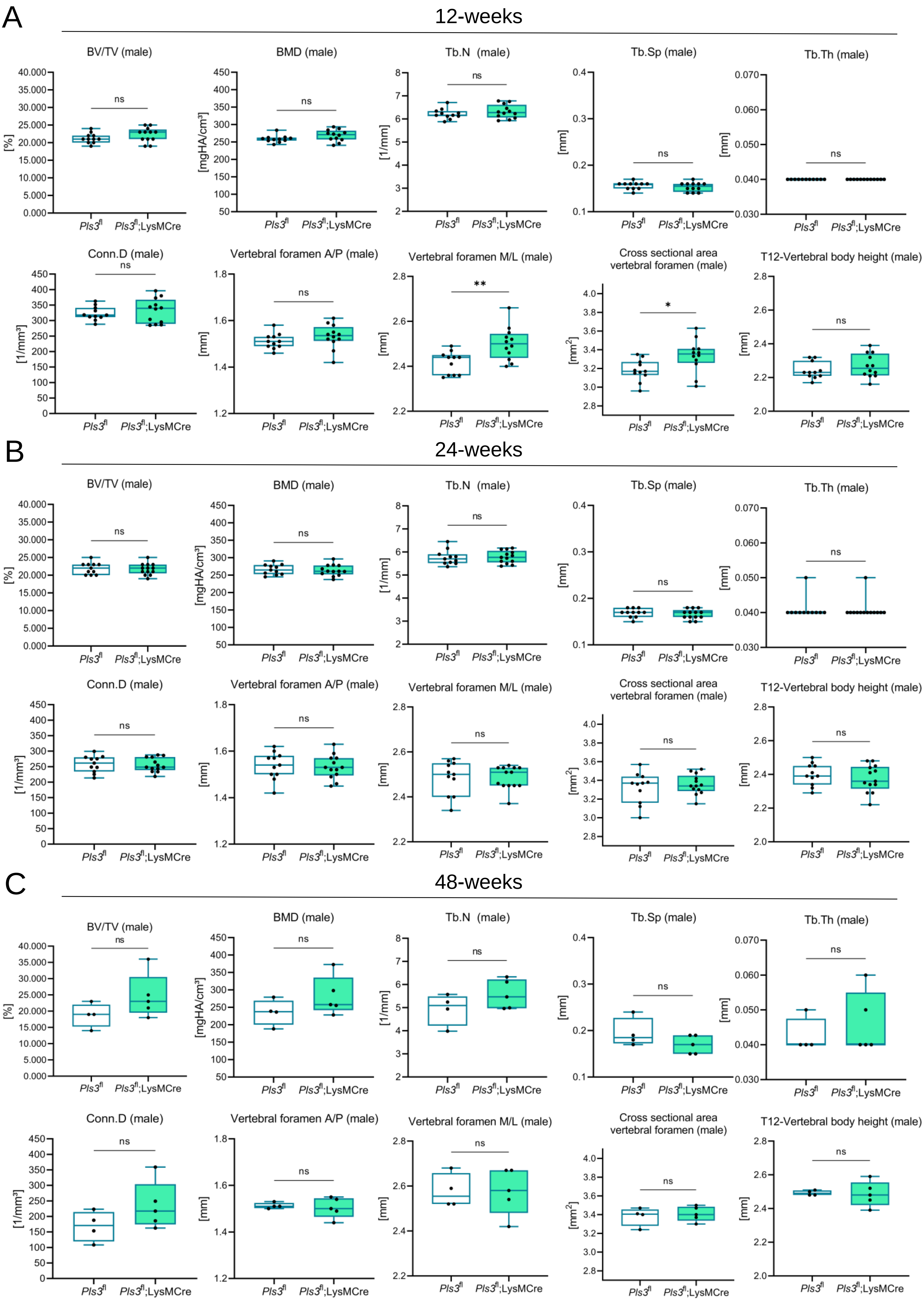
A
12-weeks
BV/TV (male)
BMD (male)
Tb.N (male)
Tb.Sp (male)
Tb.Th (male)
Conn.D (male)
Vertebral foramen A/P (male)
Vertebral foramen M/L (male)
Cross sectional area vertebral foramen (male)
T12-Vertebral body height (male)
[%]
[mgHA/cm³]
[1/mm]
[mm]
[1/mm³]
[mm²]
ns
**
*
Pls3fl
Pls3fl;LysMCre
B
24-weeks
BV/TV (male)
BMD (male)
Tb.N (male)
Tb.Sp (male)
Tb.Th (male)
Conn.D (male)
Vertebral foramen A/P (male)
Vertebral foramen M/L (male)
Cross sectional area vertebral foramen (male)
T12-Vertebral body height (male)
ns
Pls3fl
Pls3fl;LysMCre
C
48-weeks
BV/TV (male)
BMD (male)
Tb.N (male)
Tb.Sp (male)
Tb.Th (male)
Conn.D (male)
Vertebral foramen A/P (male)
Vertebral foramen M/L (male)
Cross sectional area vertebral foramen (male)
T12-Vertebral body height (male)
ns
Pls3fl
Pls3fl;LysMCre

Supplemental Fig. S10: MicroCT data of spine T12 from male *Pls3*^{fl};LysMCre$^{tg/0}$ mice in comparison to their *Pls3*^{fl} littermates. Shown are bone volume fraction (BV/TV, %), bone mineral density (BMD, mmHA/cm^3), trabecular number (Tb.N, 1/mm), trabecular separation (Tb.Sp, mm), trabecular thickness (Tb.Th, mm), and connectivity density (Conn.D, 1/mm^3), vertebral foramen A/P (mm), vertebral foramen M/L (mm), cross sectional area of the vertebral foramen (mm^2) and vertebral body height (mm) for (A) 12-, (B) for 24-, and (C) 48-week-old male mice. N = 12 of 12-week-old male *Pls3*^{fl};LysMCre$^{tg/0}$ mice; N = 11 of 12-week-old male *Pls3*^{fl} mice; N =13 of 24-week-old male *Pls3*^{fl};LysMCre$^{tg/0}$ mice; N = 11 of 24-week-old male *Pls3*^{fl} mice; N = 5 of 48-week-old male *Pls3*^{fl};LysMCre$^{tg/0}$ mice; N = 4 of 48-week-old male *Pls3*^{fl} mice. All results are shown as box plots, representing individual data points with median as a line, interquartile range (25th to 75th percentile), and min to max as whiskers. *$P < 0.05$, **$P < 0.01$, ***$P < 0.001$, ns = not significant. Statistical test: Mann-Whitney U test.

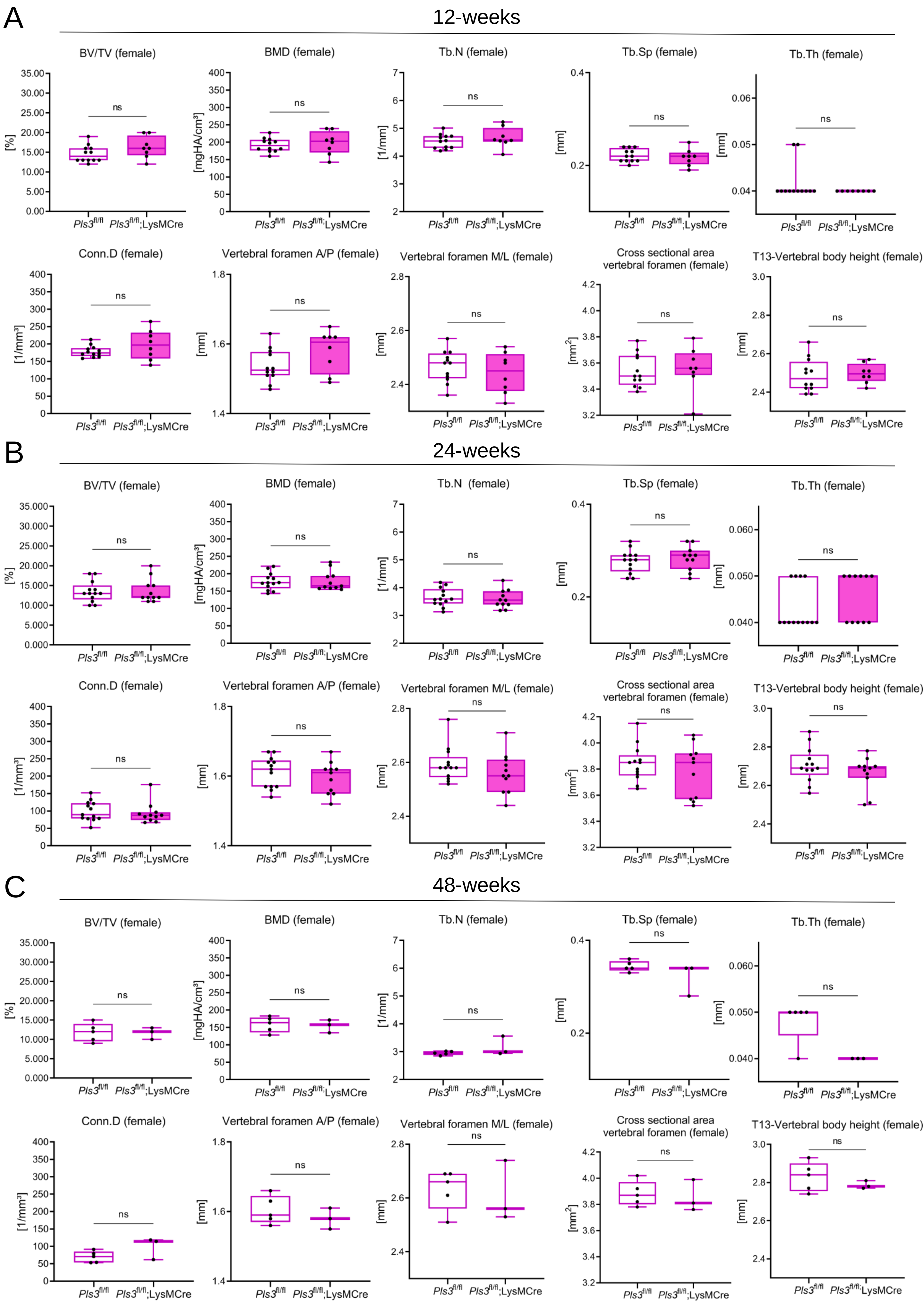
A
12-weeks
BV/TV (female)
BMD (female)
Tb.N (female)
Tb.Sp (female)
Tb.Th (female)
Conn.D (female)
Vertebral foramen A/P (female)
Vertebral foramen M/L (female)
Cross sectional area vertebral foramen (female)
T13-Vertebral body height (female)
B
24-weeks
BV/TV (female)
BMD (female)
Tb.N (female)
Tb.Sp (female)
Tb.Th (female)
Conn.D (female)
Vertebral foramen A/P (female)
Vertebral foramen M/L (female)
Cross sectional area vertebral foramen (female)
T13-Vertebral body height (female)
C
48-weeks
BV/TV (female)
BMD (female)
Tb.N (female)
Tb.Sp (female)
Tb.Th (female)
Conn.D (female)
Vertebral foramen A/P (female)
Vertebral foramen M/L (female)
Cross sectional area vertebral foramen (female)
T13-Vertebral body height (female)
[%]
[mgHA/cm³]
[1/mm]
[mm]
[1/mm³]
[mm²]
ns
Pls3fl/fl
Pls3fl/fl;LysMCre

Supplemental Fig. S11: MicroCT data of the spine T13 from female *Pls3*$^{fl/fl}$;LysMCre$^{tg/0}$ mice in comparison to their *Pls3*$^{fl/fl}$ littermates. Shown are bone volume fraction (BV/TV, %), bone mineral density (BMD, mmHA/cm^3), trabecular number (Tb.N, 1/mm), trabecular separation (Tb.Sp, mm), trabecular thickness (Tb.Th, mm), and connectivity density (Conn.D, 1/mm^3), vertebral foramen A/P (mm), vertebral foramen M/L (mm), cross sectional area of the vertebral foramen (mm^2) and vertebral body height (mm) for (A) 12-, (B) for 24-, and (C) 48-week-old female mice. N = 8 of 12-week-old female *Pls3*$^{fl/fl}$;LysMCre$^{tg/0}$ mice; N = 12 of 12-week-old female *Pls3*$^{fl/fl}$ mice; N = 11 of 24-week-old female *Pls3*$^{fl/fl}$;LysMCre$^{tg/0}$ mice; N = 13 of 24-week-old female *Pls3*$^{fl/fl}$ mice; N = 3 of 48-week-old female *Pls3*$^{fl/fl}$;LysMCre$^{tg/0}$ mice; N = 5 of 48-week-old female *Pls3*$^{fl/fl}$ mice. All results are shown as box plots, representing individual data points with median as a line, interquartile range (25th to 75th percentile), and min to max as whiskers. *$P < 0.05$, **$P < 0.01$, ***$P < 0.001$, ns = not significant. Statistical test: Mann-Whitney U test.

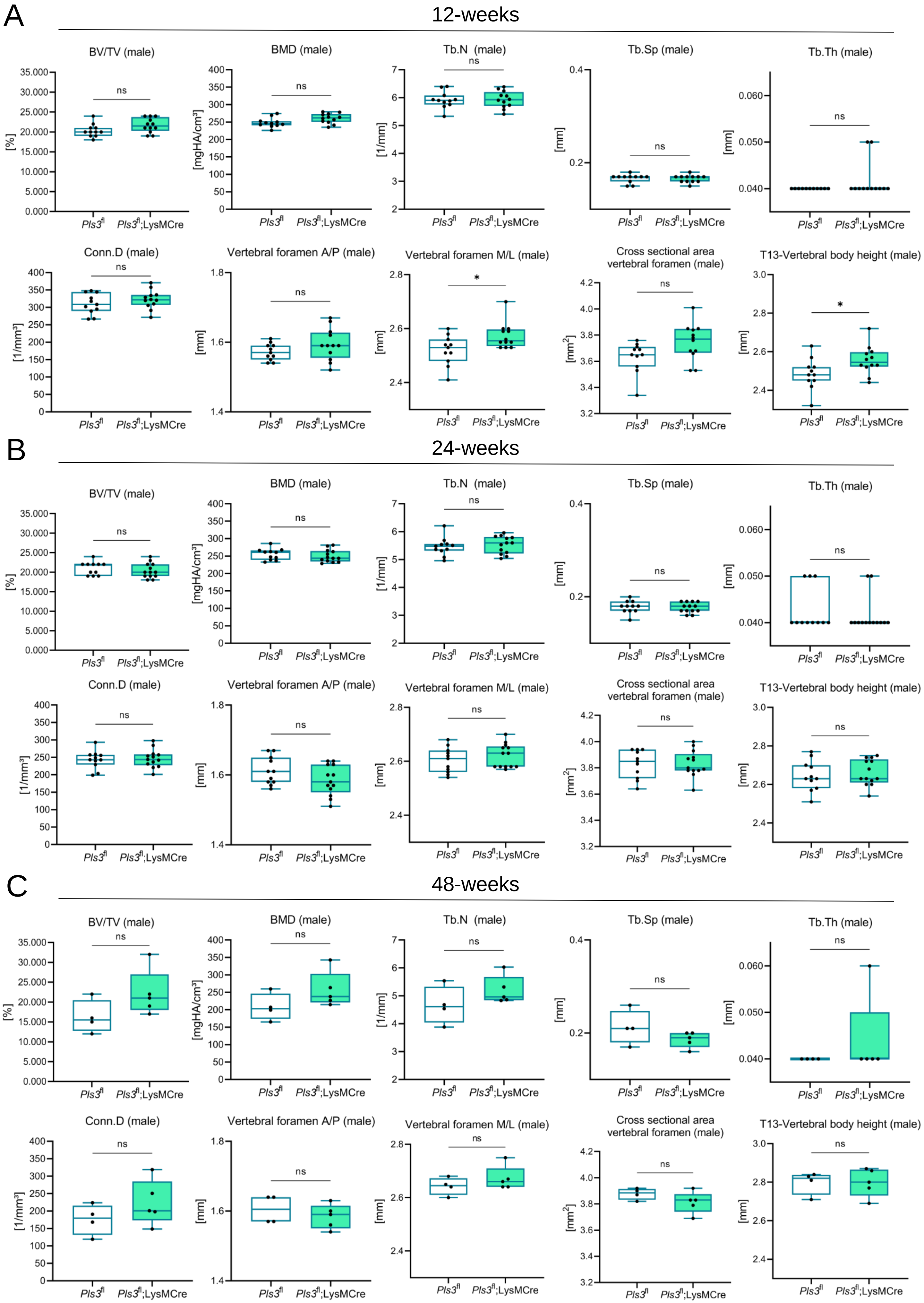

A
12-weeks
BV/TV (male)
BMD (male)
Tb.N (male)
Tb.Sp (male)
Tb.Th (male)
Conn.D (male)
Vertebral foramen A/P (male)
Vertebral foramen M/L (male)
Cross sectional area vertebral foramen (male)
T13-Vertebral body height (male)
B
24-weeks
C
48-weeks
ns
*
[%]
[mgHA/cm³]
[1/mm]
[mm]
[1/mm³]
[mm²]
Pls3fl
Pls3fl;LysMCre

Supplemental Fig. S12: MicroCT data of the spine T13 from male *Pls3*^{fl};LysMCre$^{tg/0}$ mice in comparison to their *Pls3*^{fl} littermates. Shown are bone volume fraction (BV/TV, %), bone mineral density (BMD, mmHA/cm^3), trabecular number (Tb.N, 1/mm), trabecular separation (Tb.Sp, mm), trabecular thickness (Tb.Th, mm), and connectivity density (Conn.D, 1/mm^3), vertebral foramen A/P (mm), vertebral foramen M/L (mm), cross sectional area of the vertebral foramen (mm^2) and vertebral body height (mm) for (A) 12-, (B) for 24-, and (C) 48-week-old male mice. N = 12 of 12-week-old male *Pls3*^{fl};LysMCre$^{tg/0}$ mice; N = 11 of 12-week-old male *Pls3*^{fl} mice; N =13 of 24-week-old male *Pls3*^{fl};LysMCre$^{tg/0}$ mice; N = 11 of 24-week-old male *Pls3*^{fl} mice; N = 5 of 48-week-old male *Pls3*^{fl};LysMCre$^{tg/0}$ mice; N = 4 of 48-week-old male *Pls3*^{fl} mice. All results are shown as box plots, representing individual data points with median as a line, interquartile range (25th to 75th percentile), and min to max as whiskers. *$P < 0.05$, **$P < 0.01$, ***$P < 0.001$, ns = not significant. Statistical test: Mann-Whitney U test.

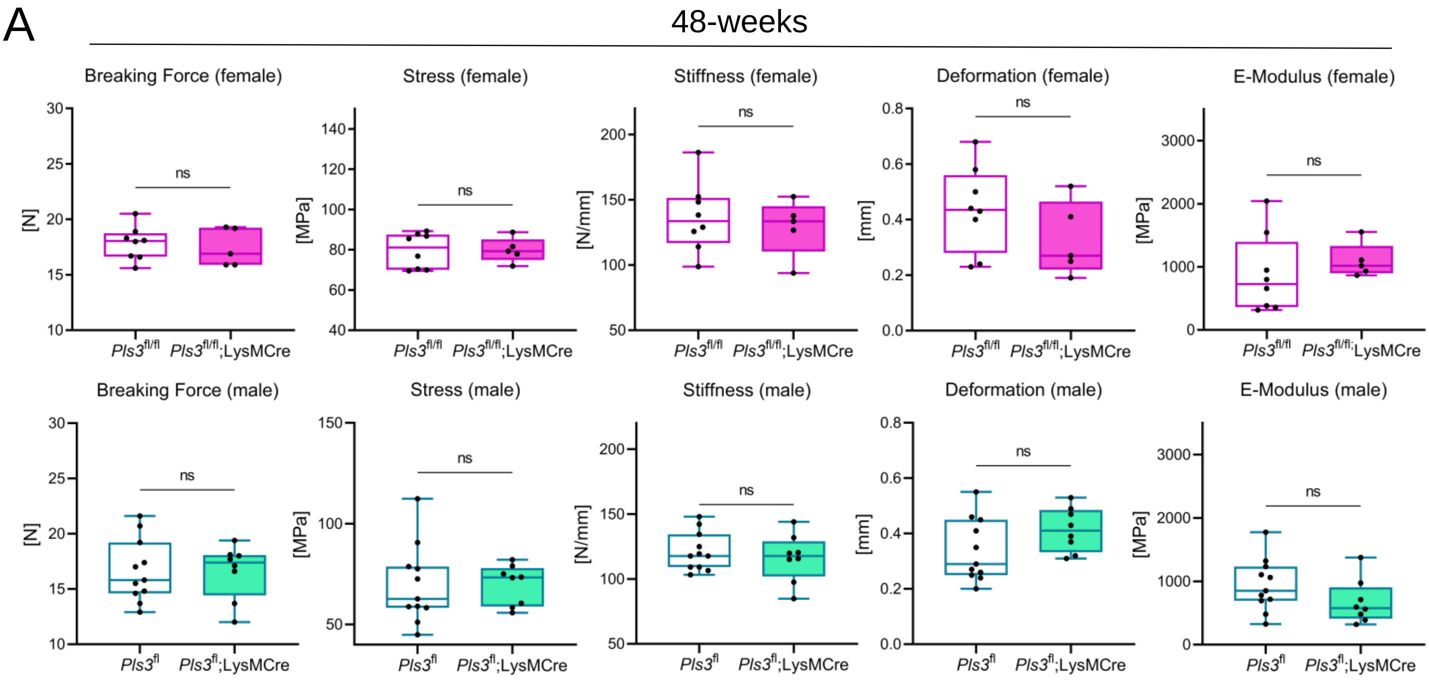

Supplemental Fig. S13: 3-point-bending-test (3-PBT) of 48-week-old female and male *Pls3*$^{fl/fl}$;LysMCre$^{tg/0}$ mice in comparison to their *Pls3*$^{fl/fl}$ littermates. Shown are breaking force, ultimate stress, stiffness, deformation, and Elastic-modulus (E-modulus). N = 5 of 48-week-old female *Pls3*$^{fl/fl}$;LysMCre$^{tg/0}$ mice; N = 8 of 48-week-old female *Pls3*$^{fl/fl}$ mice; N = 8 of 48-week-old male *Pls3*^{fl};LysMCre$^{tg/0}$ mice; N = 11 of 48-week-old male *Pls3*^{fl} mice. All results are shown as box plots, representing individual data points with median as a line, interquartile range (25th to 75th percentile), and min to max as whiskers. $*P < 0.05$, $**P < 0.01$, $***P < 0.001$, ns = not significant. Statistical test: Mann-Whitney U test.
